# Phylogenomic analyses based on the plastid genome and concatenated nrDNA sequence data reveal cytonuclear discordance in genus *Atractylodes* (Asteraceae: Carduoideae)

**DOI:** 10.3389/fpls.2022.1045423

**Published:** 2022-12-01

**Authors:** Jinxin Liu, Mengmeng Shi, Zhaolei Zhang, Hongbo Xie, Weijun Kong, Qiuling Wang, Xinlei Zhao, Chunying Zhao, Yulin Lin, Xiaoxia Zhang, Linchun Shi

**Affiliations:** ^1^ Key Laboratory of Chinese Medicine Resources Conservation, State Administration of Traditional Chinese Medicine of the People’s Republic of China, Engineering Research Center of Chinese Medicine Resource of Ministry of Education, Institute of Medicinal Plant Development, Chinese Academy of Medical Sciences & Peking Union Medical College, Beijing, China; ^2^ Hebei Key Laboratory of Study and Exploitation of Chinese Medicine, Chengde Medical University, Chengde, China; ^3^ School of Traditional Chinese Medicine, Capital Medical University, Beijing, China; ^4^ State Key Laboratory of Systematic and Evolutionary Botany, Institute of Botany, Chinese Academy of Sciences, Beijing, China

**Keywords:** *Atractylodes*, plastid genome, nrDNA concatenated sequence, phylogeny, cyto-nuclear discordance

## Abstract

*Atractylodes* species are widely distributed across East Asia and are cultivated as medicinal herbs in China, Japan, and Korea. Their unclear morphological characteristics and low levels of genetic divergence obscure the taxonomic relationships among these species. In this study, 24 plant samples were collected representing five species of *Atractylodes* located in China; of these, 23 belonged to members of the *A. lancea* complex. High-throughput sequencing was used to obtain the concatenated nrDNA sequences (18S-ITS1-5.8S-ITS2-28S) and plastid genomes. The concatenated nrDNA sequence lengths for all the *Atractylodes* species were 5,849 bp, and the GC content was 55%. The lengths of the whole plastid genome sequences ranged from 152,138 bp (*A. chinensis*) to 153,268 bp (*A. lancea*), while their insertion/deletion sites were mainly distributed in the intergenic regions. Furthermore, 33, 34, 36, 31, and 32 tandem repeat sequences, as well as 30, 30, 29, 30, and 30 SSR loci, were detected in *A. chinensis*, *A. koreana*, *A. lancea*, *A. japonica*, and *A. macrocephala*, respectively. In addition to these findings, a considerable number of heteroplasmic variations were detected in the plastid genomes, implying a complicated phylogenetic history for *Atractylodes*. The results of the phylogenetic analysis involving concatenated nrDNA sequences showed that *A. lancea* and *A. japonica* formed two separate clades, with *A. chinensis* and *A. koreana* constituting their sister clade, while *A. lancea*, *A. koreana, A. chinensis*, and *A. japonica* were found based on plastid datasets to represent a mixed clade on the phylogenetic tree. Phylogenetic network analysis suggested that *A. lancea* may have hybridized with the common ancestor of *A. chinensis* and *A. japonica*, while ABBA–BABA tests of SNPs in the plastid genomes showed that *A. chinensis* was more closely related to *A. japonica* than to *A. lancea*. This study reveals the extensive discordance and complexity of the relationships across the members of the *A. lancea* complex (*A. lancea, A. chinensis*, *A. koreana*, and *A. japonica*) according to cytonuclear genomic data; this may be caused by interspecific hybridization or gene introgression.

## Introduction

*Atractylodes*, mainly distributed across East Asia, is a perennial herb from the Asteraceae family. The dried rhizomes of *Atractylodes* plants have been used for more than 2,000 years as traditional herbal medicines (“cangzhu” and “baizhu” in China, and so-jutsu and byaku-jutsu in Japan) to treat gastroduodenal diseases and colds (Yin et al., 2015; Deng et al., 2016). Due to the limitations of wild resources, the plants of *Atractylodes* have been cultivated in China since the 1980s, and production of their dried rhizome has now reached 5000 tons per year. However, the cultivation of particular species of *Atractylodes* presents the challenge of heterogeneous germplasm, which is mainly caused by high variability and continuous variation in morphological features among individual plants (Deng et al., 2016). In 1959, *A. chinensis* DC. was considered an independent species in Northeast Medicinal Flora (Liu, 1959) and was divided into several variants, such as *A. chinensis* var. *koreana* (Nakai) Chu, *A. chinensis* DC. var. *simplicifolia* (Loes.) Chu, and *A. chinensis* DC. var. *liaotungensis* Kitag. In 1981, Shi (Zhu, 1981) considered pinnatipartite leaves to be a volatile mutation, and further claimed, following an in-depth analysis of documented specimens and the literature, that the labels *A. chinensis* and *A. lancea* were actually used to refer to a single species. In 1987, *A. chinensis* was treated as a synonym of *A. lancea* according to the law of priority nomenclature in Flora Reipublicae Popularis Sinicae (Lin and Shi, 1987). In 2011, according to Flora of China, *A. japonica* was also treated as a synonym of *A. lancea;* at present, *A. lancea* is an overcrowded group whose leaves are described as undivided or divided almost to base into 3-5(-9) pinnately arranged segments (Shi, 2011). The nomenclatural history of *Atractylodes* is illustrated in **Supplementary Figure 1**. Although several molecular markers have been used to investigate the taxonomic and phylogenetic characteristics of *Atractylodes* (Peng et al., 2012; Kim et al., 2016), there is still taxonomic controversy surrounding this genus, arising primarily from the interspecific and intraspecific taxonomic treatment of the *A. lancea* complex, consisting of *A. japonica*, *A. koreana*, *A. lancea*, and *A. chinensis*.

The rapid development of next-generation sequencing (NGS) technology (McPherson et al., 2013), coupled with powerful bioinformatic tools (Liu et al., 2012; Shi et al., 2019), has made it possible to study the genomic evolution and interspecific relationships of organisms according to whole plastid genomic data. Substantial nucleotide substitution, indel events, and structural rearrangements have been found in plastid genomes, indicating that the whole plastid genome contains a significant amount of phylogenetic information (Liu et al., 2018). Additionally, plant plastids display intraspecific heteroplasmic variation, a term which refers to the presence of nonidentical plastid molecules in a cell or organism (Scarcelli et al., 2016). Previous studies have used NGS methods to detect heteroplasmy in the plastid genome of *Astragalus membranaceus*, which could explain why the *de novo* genome assembly program has failed to assemble the genome in heterogeneous regions (Lei et al., 2016). Moreover, intra-individual polymorphism can provide new evidence that can be used in evolutionary and classification analysis (Sun et al., 2019). In addition, extensive phylogenetic discordance among nuclear and organellar phylogenies has been found in the genus *Sphagnum*; this has been caused by incomplete lineage sorting (ILS) following the rapid radiation of the genus, rather than by post-speciation introgression (Meleshko et al., 2021).

In 2020, Wang et al. used the plastomes and nuclear sequences of *Atractylodes lancea*, *A. chinensis*, and *A. macrocephala* to reconstruct the phylogenetic relationships of these three species (Wang et al., 2020). Phylogeny analysis using the plastid data indicated that *A. lancea* and *A. chinensis* are more closely related to one another than to *A. macrocephala*. Interestingly, this study further observed intra-individual polymorphism of SLD5, such that SLD5 has two haplotypes in *A. macrocephala*, of which one can be found in *A. lancea* and, separately, the other can be found in *A. chinensis*. This intra-individual polymorphism was taken to imply that *A. macrocephala* may be a hybrid of *A. lancea* and *A. chinensis*, or the result of introgressive hybridization (Wang et al., 2020). Subsequently, analysis of six plastid genomes of *Atractylodes* (*A. chinensis*, *A. koreana*, *A. lancea*, *A. macrocephala*, *A. japonica*, and *A. carlinoides*) has revealed that the phylogenetic relationship within *Atractylodes* is complex (Wang et al., 2021). The results indicated that *A. japonica* and *A. lancea* are clustered into a subclade, while *A. chinensis* and *A. koreana* are clustered into another subclade. The abovementioned studies have confirmed that plastid genome analysis is a valuable tool for the phylogenetic study of *Atractylodes*, and additional specimens should be collected to obtain further evidence on the complex evolutionary history of *A. lancea* (Wang et al., 2021).

In this study, we collected 24 plant samples, 23 of which represented species of *A. lancea* complex. High-throughput sequencing was used to obtain the concatenated nrDNA sequences (18S-ITS1-5.8S-ITS2-28S) and plastid genomes. Our analyses showed that *A. chinensis* was more closely related to *A. japonica* than to *A. lancea*. Furthermore, extensive discordance and complex relationships across the genus of *Atractylodes* were revealed through analysis of the cytonuclear genomic data.

## Materials and methods

### Sample collection

For this study, 24 samples representing specimens of *Atractylodes lancea*, *A. chinensis*, *A. macrocephala*, *A. japonica*, and *A. koreana* were collected, of which six samples were collected from wild regions and 18 samples from cultivated regions (**Supplementary Figure 2**). All the samples were morphologically authenticated by Chunying Zhao (Chengde Medical University), Xinlei Zhao (Institute of Medicinal Plant Development, CAMS), Yulin Lin (Institute of Medicinal Plant Development, CAMS), and Qiuling Wang (Institute of Medicinal Plant Development, CAMS). Detailed information is provided in **Supplementary Table 1** and **Supplementary Figure 3**. In addition, data relating to 20 samples representing six species of genus *Atractylodes* and ten species of outgroup taxa were downloaded from GenBank (**Supplementary Table 2**).

### DNA extraction, library preparation, and high-throughput sequencing

Total genomic DNA extraction was performed on the leaf tissues using a modified CTAB method. The quantity and quality of the DNA were determined using Qubit 4.0 (Thermo Fisher Scientific Inc., USA). The sequencing library (~350 bp) was constructed using purified DNA and a TruSeq DNA PCR-Free High Throughput Library Prep Kit (Illumina USA). An Illumina NovaSeq platform was employed to conduct high-throughput sequencing. The raw data were deposited in the Sequence Read Archive (SRA) under BioProject accession number PRJNA682118. The final plastid genomes and concatenated nrDNA sequences of the *A. lancea*, *A. chinensis*, *A. macrocephala*, *A. japonica*, and *A. koreana* specimens were assembled, annotated, and submitted to GenBank (**Supplementary Table 1**).

### Assembly, annotation, and characterization of the concatenated nrDNA and plastid genome sequences

The sequencing adapter and low-quality reads were filtered using Trimmomatic v0.38 (Bolger et al., 2014). Whole plastid genomes were assembled *via* the organelle assembler NOVOPlasty v4.2.1 (Jin et al., 2020) and GetOrganelle (Jin et al., 2020). The plastid genome sequence of *A. lancea* (accession number: NC_037483) was selected as a reference in the NOVOPlasty configuration file. CpGAVAS2 (www.herbalgenomics.org/cpgavas2) with default parameters was used to annotate the protein-coding, rRNA, and tRNA genes of the plastid genome and to facilitate visualization (Shi et al., 2019), with the initial annotations being edited manually using the Apollo genome editor. A circular map was generated using OrganellarGenomeDRAW (OGDRAW) (Greiner et al., 2019). The concatenated nrDNA sequences (18S, ITS1, 5.8S, ITS2, and 28S nrDNA) were assembled using Getorganelle and compared with the nuclear ribosomal RNA database to obtain the annotation results. The codon usage and relative synonymous codon usage (RSCU) of the plastid genomes were calculated using CodonW (http://codonw.sourceforge.net/).

### Analysis of repeat structures and intraspecific variation in the plastid genomes

The REPuter (Kurtz et al., 2001) program was used to identify four types of sequence repeats, including forward (F), reverse (R), complementary (C), and palindromic (P). The minimum repeat size for oligonucleotide repeats was set at 30 bp, with a Hamming distance of 3 (i.e., a sequence identity of 90%). Tandem repeats were analyzed using the TRF (Benson, 1999) software with default parameters. Simple sequence repeats (SSRs) were detected using the MIcroSAtellite identification tool (MISA, available online: http://pgrc.ipk-gatersleben.de/misa/) (Beier et al., 2017) with minimum repeat thresholds of 10, 6, 5, 5, 5, and 5 for mono-, di-, tri-, tetra-, penta-, and hexa-nucleotide SSRs, respectively. Intraspecific variations were detected by first mapping the reads to reference sequences using Bowtie2 (Langmead and Salzberg, 2012), and subsequently conducting analysis using our local python program and visualizing the sequences using an integrative genomics viewer.

### Comparative analysis of the plastid genomes

The mVISTA program (http://genome.lbl.gov/vista/mvista/submit.shtml) was used in Shuffle-LAGAN mode for the comparative analysis of divergence regions with default parameters, and *A. chinensis* was used as a reference. Intra- and inter-distances were analyzed in accordance with procedures reported in our previous study (Chen et al., 2010). Mauve (Darling et al., 2004), a system used to construct multiple genome alignments in the presence of large-scale evolutionary events, was used to identify locally collinear blocks (LCBs) of *Atractylodes* species. The contraction and expansion of the IR boundaries between the four main parts of the genome (LSC/IRb/SSC/IRa) were visualized using IRscope (https://irscope.shinyapps.io/irapp/).

### Phylogenetic and gene introgression analysis

A total of 44 Asteraceae whole plastid genomes and concatenated nrDNA sequences were used for phylogenetic analysis. *Lactuca raddeana* and *Ainsliaea latifolia* were used as the outgroup species (**Supplementary Table 2**). Each region was first aligned using MUSCLE v3.8 (Edgar, 2004) and then concatenated to form six matrices, namely 1) a dataset of concatenated nrDNA sequences (aligned length 5,855 bp), 2) a dataset of 73 conserved protein-coding sequences (aligned length 61,413 bp), 3) a dataset of 95 common genes including rRNA and tRNA genes (aligned length 83,547 bp), 4) a dataset of 88 intergenic spacer regions (IGS, aligned length 37,809 bp), 5) a dataset of 73 protein sequences (aligned length 20,393 bp), and 6) the dataset of the whole plastid genomes (aligned length 121,356 bp). The maximum likelihood (ML) phylogenetic trees of the six matrices were constructed using RAxML v8.2.12 (Stamatakis, 2014) with 1000 bootstrap replicates. The GTRGAMMA substitution model was applied to the protein-coding genes, genes, IGS, and whole plastid genomes, while PROGAMMAAUTO was applied to protein sequences. The parameters for this analysis included “raxmlHPC-PTHREADS-SSE3 -f a -N 1000 -m GTRGAMMA -x 551314260 -p 551314260 -T 20”. Tree visualization was performed using MEGA X (Kumar et al., 2018). Finally, the topologies recovered after analysis of the plastid and nrDNA data were compared using the dendextend package (Galili, 2015).

The analysis of phylogenetic networks was carried out using PhyloNet v.3.8.21 (Wen et al., 2018) with the command ‘InferNetwork_MPL’ using gene tree topologies estimated by IQ-TREE2 (Minh et al., 2020); subsequently, the phylogenetic networks in the form of Rich Newick strings were visualized in Dendroscope3 (Huson and Scornavacca, 2012). The D-statistic (ABBA–BABA) method in Dsuite (Malinsky et al., 2021) was used to test for introgression events using SNP data on *Atractylodes* plastid genomes.

## Results

### The structure of the *Atractylodes* plastid genome and concatenated nrDNA sequences

**Figure 1:**
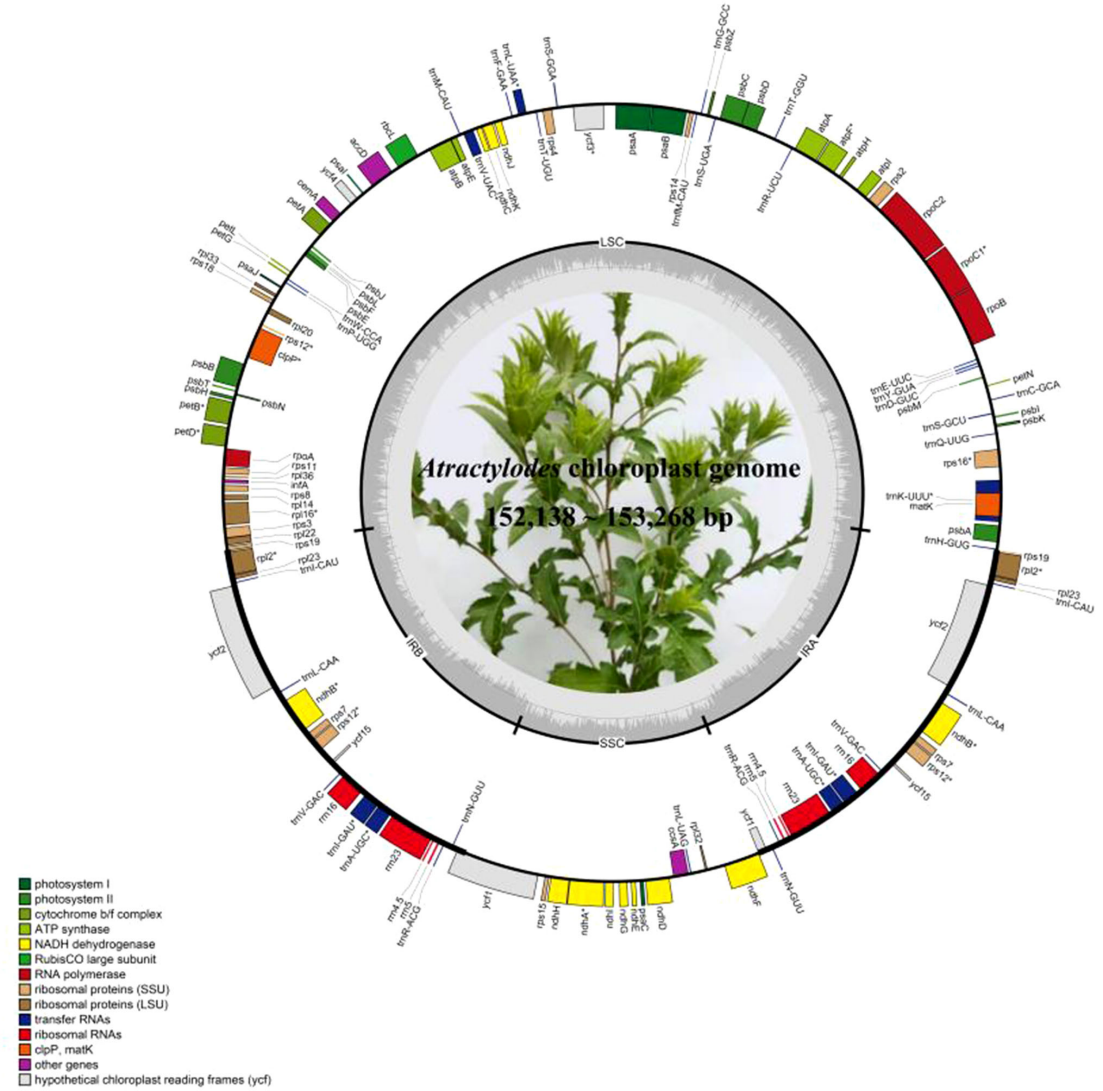
Plastid genome map of *Atractylodes* species. Gene locations outside the outer rim are transcribed in a counterclockwise direction, while genes inside are transcribed in a clockwise direction. The colored bars indicate known functional family differences. The dashed gray area in the inner circle shows the proportional GC content of the corresponding genes. LSC, large single-copy region; SSC, small single-copy region; IR, inverted repeat region.

The plastid genome length of *Atractylodes* ranged from 152,138 bp (*A. chinensis*) to 153,268 bp (*A. lancea*). The greatest length variations among individuals of *A. chinensis*, *A. koreana*, and *A. lance*a were 956 bp, 983 bp, and 1000 bp, respectively. Each plastid genome displayed a typical quadripartite structure, consisting of a large single-copy (LSC) region (83,206~84,293 bp), a small single-copy (SSC) region (18,604~18,698 bp), and a pair of inverted repeat (IR) regions (IRa and IRb) (25,137~25,185 bp). The GC content varied from 37.69% to 37.77% and was higher in the IR regions (about 43%) than in the LSC and SSC regions (about 35% and 31%) in all species (**Figure 1**, **Supplementary Table 3**). Moreover, several long-term indels were verified by genome mapping, including the 989 bp deletion of the plastid *ndhC_trnV-UAC* IGS in *A. lancea* (HPAB0031), *A. chinensis* (HPAB0001, HPAB0003, and HPAB0006), and *A. koreana* (HPAB0010). Length differences in coding genes such as *rpoB* and *ycf2* were mainly due to the occurrence of repeat units in the sequence. A 6 bp insertion unit (TTAACC) of *rpoB* was found in *A. lancea* (HPAB0027), while a 9 bp deletion unit of *ycf2* was present in *A. chinensis* (HPAB0001).

A total of 113 unique genes were annotated in the plastid genomes, consisting of 80 protein-coding genes, 29 tRNA genes, and four rRNA genes (*rrn23S*, *rrn16S*, *rrn5S*, and *rrn4.5S*). Of these, 82 genes (61 protein-coding genes and 21 tRNA genes) were located in the LSC region. The SSC region contained 12 protein-coding genes and one tRNA gene (*trnL-UAG*). Furthermore, 17 genes contained introns: 14 (nine protein-coding and five tRNA genes) contained one intron, and three (*rps12*, *ycf3*, and *clpP*) contained two introns (**Supplementary Table 4**). Small exons were also annotated in the *petB*, *petD*, and *rpl16* genes, with lengths of 6 bp, 8 bp, and 9 bp, respectively. Finally, *rps12* was identified as a trans-splicing gene.

The length of the concatenated nrDNA sequence was 5,849 bp; this sequence consisted of five parts. The lengths of 18S, ITS1, 5.8S, ITS2, and 28S were 1,809 bp, 259 bp, 158 bp, 229 bp, and 3,394 bp, respectively. The GC content varied between 55.43% and 55.62% and was higher in the ITS regions (about 63%, including ITS1 and ITS2) than in the 18S and 28S regions (about 49% and 57%) in all samples (**Supplementary Table 5**).

### Codon usage in the *Atractylodes* plastid genomes

The amino acid frequency, codon usage, and relative synonymous codon usage (RSCU) of 80 protein-coding regions in all *Atractylodes* species were analyzed using Codon W. RSCU values ranged from 63.98 to 64.05; the number of codons ranged from 22,846 (HPAB0022) to 22,873 (HPAB0023) in 26 species; and the number of amino acids ranged from 21,706 (HPAB0023) to 22,398 (HPAB0016). Of these codons, leucine (2,275 ~ 2,338 codons) was the most abundant amino acid, with a frequency of 9.95 ~ 10.22%, while the proportion of cysteine (471 ~ 486 codons) was 2.06 ~ 2.13%; AGA (encoding arginase) and CGC (encoding arginase) were the most and least used codons, respectively (**Supplementary Table 6**). Almost all the amino acids had more than one synonymous codon; the exceptions were methionine and tryptophan. Furthermore, 31 codons displayed RSCU values exceeding 1. Most of the biased codons were used with A or T bases as the third codons. ATG and TGG, encoding methionine and tryptophan, exhibited no bias (RSCU = 1.00) (**Supplementary Table 6**).

Three types of starting codons were detected in 80 protein-coding genes. Of these, 77 genes used ATG as start codons, while two (*ndhD*, *psbL*) used ACG and one (*rps19*) used GTG. TAA, TAG, and TGA were present as stop codons in these genes. The most used stop codon was TAA at 55.56%, followed by TAG (25.92%) and TGA (18.52%). *ndhF* was the only gene that used both TAA and TGA as stop codons; of this gene, eight samples (HPAB0003, HPAB0006, HPAB0009, HPAB0010, HPAB0011, HPAB0016, HPAB0018, and HPAB0031) showed a preference for TAA and 18 utilized TGA.

### The SSR units and repeat structures of *Atractylodes* plastid genomes

SSRs were detected in 24 *Atractylodes* plastid genome sequences (**Supplementary Table 7**). Specifically, 30, 30, 29, 30, and 30 SSR loci were detected in the *Atractylodes chinensis*, *A. koreana*, *A. lancea*, *A. japonica*, and *A. macrocephala* plastid genomes, respectively. A large proportion of the SSRs were distributed in the LSC region (85.23%), with 12 in the SSC region and 10 in the IR regions. Polyadenine (poly-A) (34.70%, 10~16) and polythymine (poly-T) (60.41%, 10~21) represented the dominant repeats.

**Figure 2:**
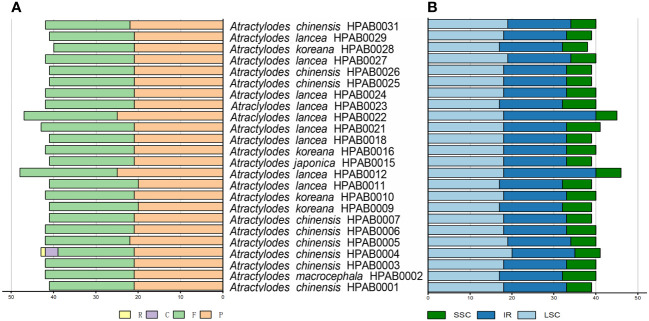
The types and distribution of repeat sequences in 24 *Atractylodes* plastid genomes. **(A)** The number of each of the four repeat types (F, forward; P, palindrome; R, reverse; C, complement). **(B)** The distribution of repeat sequences across three regions: LSC, SSC, and IR.

In addition to the SSRs, 33, 34, 36, 31, and 32 tandem repeat sequences were detected in the *A. chinensis*, *A. koreana*, *A. lancea*, *A. japonica*, and *A. macrocephala* plastid genomes, respectively. The tandem repeat lengths were 9~45 bp, and most were located in the IGS regions of the genomes. In addition, 1, 3, 5, 5, 5, 47, and 25 tandem repeats were found in the *atpI*, *ndhF*, *rpoC2*, *rps18*, *petD*, *ycf1*, and *ycf2* coding regions, respectively (**Figure 2**, **Supplementary Table 8**). Finally, an average of 42 repeat structures were revealed for each species, including F, P, R, and C repeats. P was the most common repeat type, accounting for 48.8~53.2% of all repeats, followed by F (41.9~51.2%), C (6.9%), and R (2.3%) (**Figure 2**, **Supplementary Table 9**).

### Variation in *Atractylodes* plastid genome sequences and concatenated nrDNA sequences

**Figure 3:**
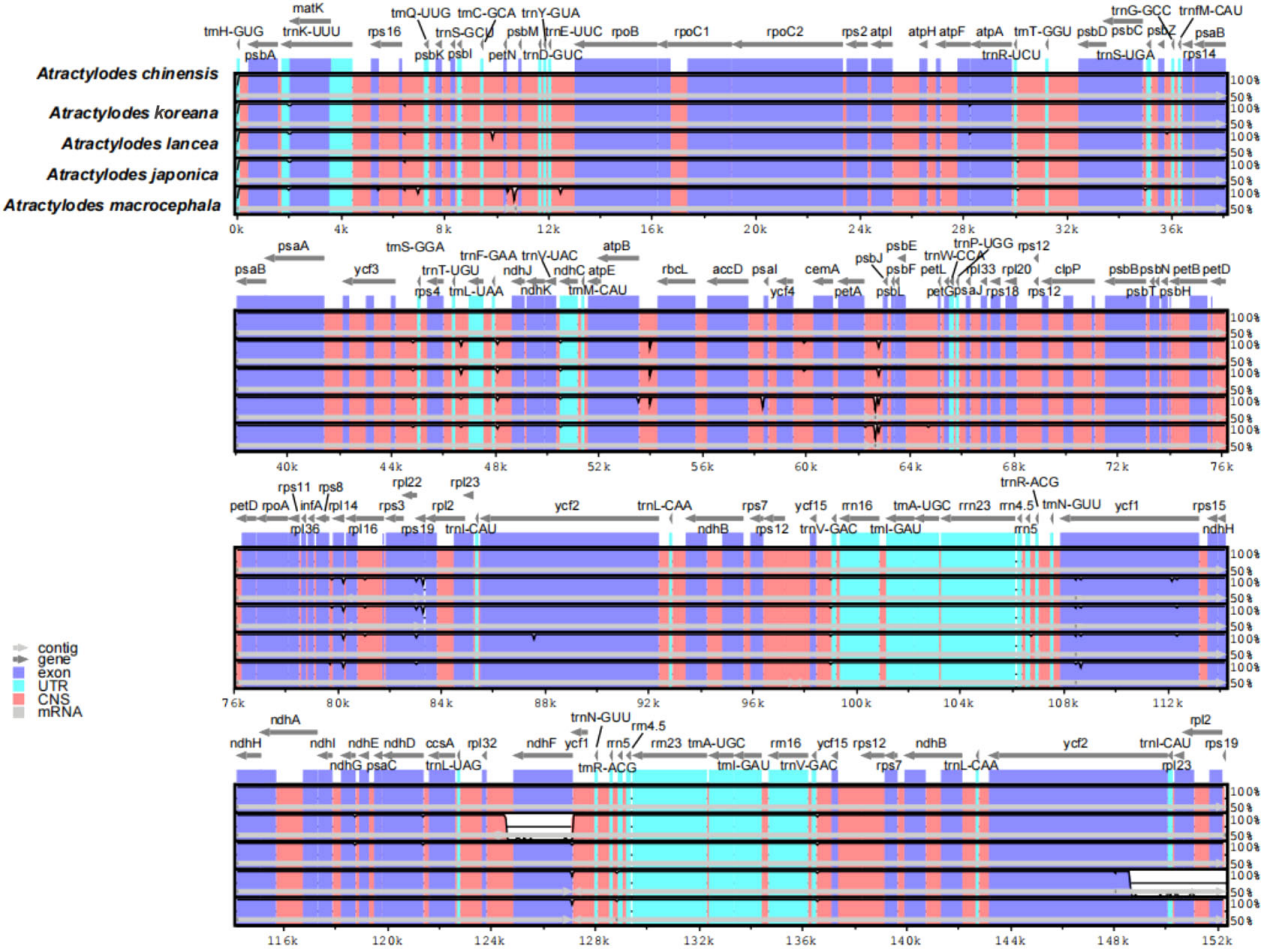
A comparison of the five plastid genomes, with *A. chinensis* as a reference, using the mVISTA alignment program. The grey arrows above the alignments show the orientation of the genes. The violet blocks indicate exons, the cyan blocks denote introns, and the salmon blocks signify conserved non-coding sequences (CNS). The y-axis indicates the identity percentage, ranging from 50% to 100%, while the x-axis represents sequence length.

**Figure 4:**
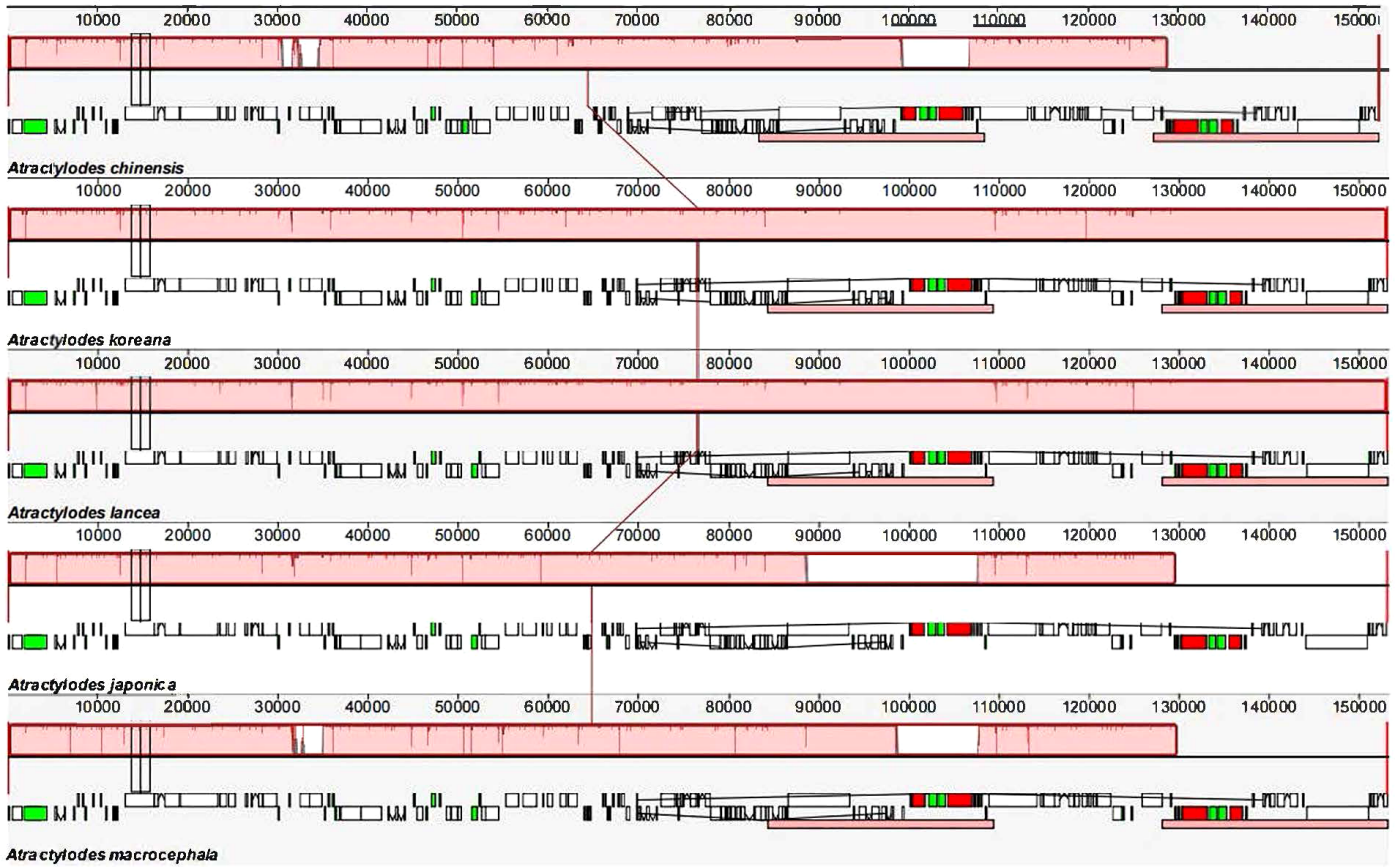
A comparison of the whole plastid genomes of the *Atractylodes* species using the Mauve algorithm. The red LCBs indicate syntenic regions, while the histograms within each block represent the degree of sequence similarity. rRNA, protein-coding, and tRNA gene annotations are denoted by red, white, and green boxes, respectively.

The 24 plastid genome sequences were compared to identify differences using the online software platform mVISTA, with the *A. chinensis* plastome as the reference genome (**Figure 3**, **Supplementary Figure 4**). Highly conservative regions were evident across most of the plastid genomes, and only three relatively high-variability regions were identified in the whole genome (**Figure 3**). Subsequently, Mauve was used to identify local collinear blocks (LCBs) of *Atractylodes* plastid genomes (**Figure 4**, **Supplementary Figure 5**); the *A. chinensis* genome is shown at the top as the reference genome. These species showed a consistent sequence order in all the genes. The collinear blocks of all the plastid genomes, including the LSC, SSC, and IR regions, revealed relatively high levels of conservation, with no gene rearrangement.

The nrDNA sequences exhibited 53 variation sites and 25 parsimony-informative sites, accounting for 0.91% and 0.43% of the total nrDNA sequences, respectively. The majority of the variation sites were located in the ITS1 and ITS2 regions. The average inter-specific distance, average theta prime, and smallest inter-specific distance were used to characterize the inter-specific divergence, taking values of 0.0027, 0.0027, and 0.0014, respectively. The intra-specific variation was determined according to the average intra-specific difference, theta, and average coalescent depth, which yielded values of 0.0003, 0.0008, and 0.0012, respectively. DNA barcoding gaps were clearly present for species *A. carlinoides*, *A. macrocephala*, *A. japonica*, and *A. lancea*, whereas the relationship between *A. chinensis* and *A. koreana* could not be resolved.

### Contraction and expansion of the IR region in *Atractylodes* plastid genome sequences

**Figure 5:**
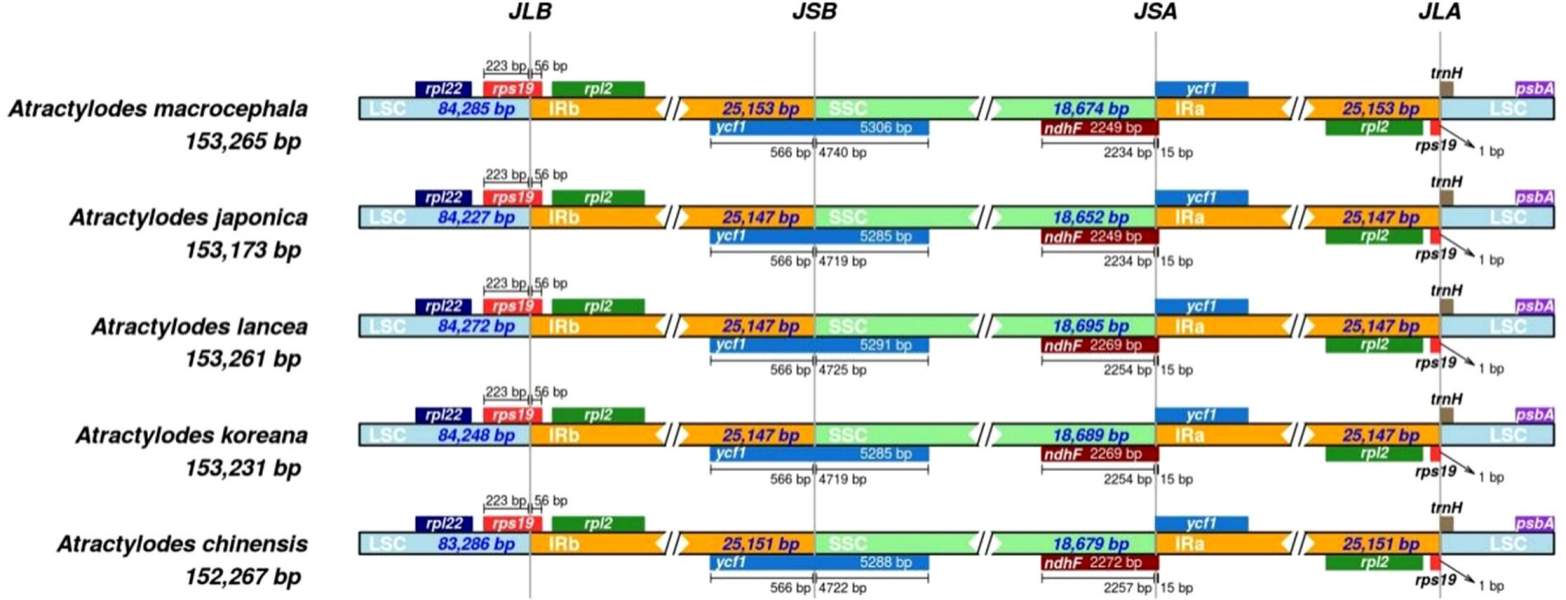
A comparison of the LSC and IRb border region and the SSC and IRa border region for the five *Atractylodes* species. JLB: junction of LSC and IRb; JSA: junction of SSC and IRa. JSB: junction of IRb and SSC; JLA: junction of IRa and LSC.

The circular structure of the plastid genome was highly conserved and generated four boundaries in the IR, LSC, and SSC regions. As the genome evolved, the contraction and expansion of the IR boundary produced different plastid genome sizes and altered certain gene locations. The *rps19* gene crossed the LSC and IRb regions in all the species. Although the *ycf1* gene was distributed across the SSC and IRb regions, most of the genes were located in the SSC region, with minor differences in length. Four indels were identified *via* multiple sequence alignment in the *ycf1* gene. Specifically, a TTTGAA insertion was detected in positions 4,435-4,440 in *A. chinensis* and *A. macrocephala*; an AAGACGAA deletion occurred at positions 800-808 in *A. chinensis*; an AAATAC deletion was evident at positions 4,290-4,295 in *A. lancea*; and finally, an AAGACGAAG insertion was detected at positions 791-799 in *A. macrocephala*. The *ndhF* gene and the *ycf1* pseudogene were detected at the junction of SSC and IRa. The *ndhF* gene was mainly located in the SSC region but spanned the junction 15 bp into the IRB region. The *ycf1* pseudogene was entirely located in the IRa region. Additionally, the *rps19* pseudogene and *trnH* gene were located at the junction of the IRa and LSC regions, while *rps19* spanned the junction 1 bp into the LSC region (**Figure 5**, **Supplementary Figure 6**).

### Heteroplasmic variations in the whole plastid genome

A total of 64 heteroplasmic variations were detected in the whole plastid genome of *Atractylodes* (**Supplementary Table 10**). This heteroplasmy consisted primarily of two types, namely heteroplasmy in insertion/deletions (indels) and heteroplasmy in single nucleotide polymorphism sites (SNPs). For example, an *ndhF* gene sequence deletion of 20 bp in length was identified in eight samples (HPAB0003, HPAB0006, HPAB0009, HPAB0010, HPAB0011, HPAB0016, HPAB0018, and HPAB0031) *via* multiple sequence alignment. Further genome mapping simultaneously detected two types of reads (insertion and deletion) in the intra-plastid genome, such as in sample HPAB0016. A further example is that the base R representing adenine (A) and guanine (G) was found in the assembly result for HPAB0001. Subsequently, a total of 235 reads related to this region were extracted from the shotgun sequencing data, indicating the simultaneous detection of G (185) and A (50) at the corresponding site (**Supplementary Table 10**).

### Phylogenetic tree, phylogenetic network, and gene introgression analyses

The phylogenetic relationships of *Atractylodes* were analyzed using six data sets of concatenated nrDNA sequences, 73 conserved plastid protein-coding gene sequences, 95 plastid common gene sequences, 88 plastid IGS regions, 73 plastid protein sequences, and the whole plastid genome sequences (**Supplementary Table 11**). The resulting phylogenetic trees showed that *Atractylodes* was a monophyletic clade related to *Tugarinovia mongolica*. The concatenated nrDNA sequences and plastid phylogenetic analyses indicated that *A. carlinoides* separated from the rest of the *Atractylodes* species with a high bootstrap value. *A. macrocephala* alone formed a relatively independent clade, with the *A. lancea* complex as a sister group of this.

**Figure 6:**
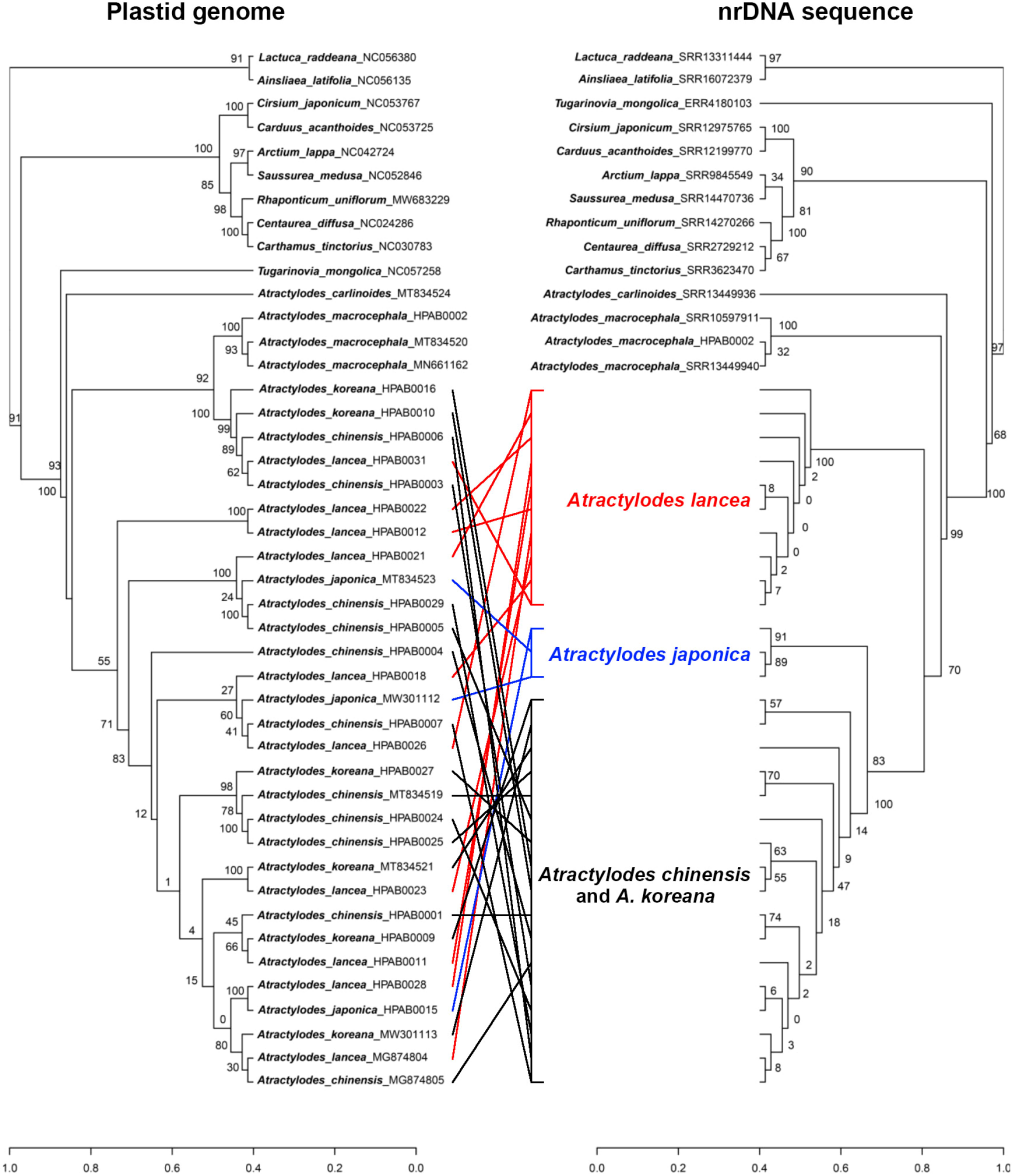
A comparison of the topologies recovered *via* plastid data (left) and *via* concatenated nrDNA sequences (right). *A. lancea* are shown in red*, A. japonica* in blue, and the *A. chinensis–A. koreana* clade in black.

The phylogenetic tree generated for the concatenated nrDNA sequence dataset showed that the *A. lancea* complex was divided into three subclades: *A. lancea*, *A. japonica*, and *A. chinensis–A. koreana*. The *A. lancea* clade included ten samples (HPAB0011, HPAB0012, HPAB0018, HPAB0021, HPAB0022, HPAB0023, HPAB0026, HPAB0028, HPAB0031, and MG874804). The *A. japonica* clade included three samples (HPAB0015, MW301112, and MT834523), which were outside the *A. chinensis–A. koreana* clade with a bootstrap value of 91. The most controversial aspect of the tree was the *A. chinensis–A. koreana* clade, which included 17 A*. chinensis* and *A. koreana* samples (**Figure 6**, **Supplementary Figure 7**).

The phylogenetic trees for the five different plastid datasets presented similar topologies (**Supplementary Figures 8**-**Supplementary Figure 12**). The whole plastid genome dataset yielded better-supported trees than the other four datasets. In this tree, the *A. lancea* complex was divided into one small and one large clade. The small clade was a sister of *A. macrocephala* and contained two *A. chinensis* samples (HPAB0003 and HPAB0006), one *A. lancea* sample (HPAB0031), and two *A. koreana* samples (HPAB0010 and HPAB0016) with a bootstrap value of 100. The remaining 25 samples, consisting of nine *A. lancea*, three *A. japonica*, four *A. koreana*, and nine *A. chinensis*, formed a large clade with a bootstrap value of 55. The phylogeny of the four species in this large clade was ambiguous and could not be clearly resolved (**Figure 6**). Phylogenetic analyses for the nrDNA and whole plastid genome indicated plastid and nuclear discordance.

**Figure 7:**
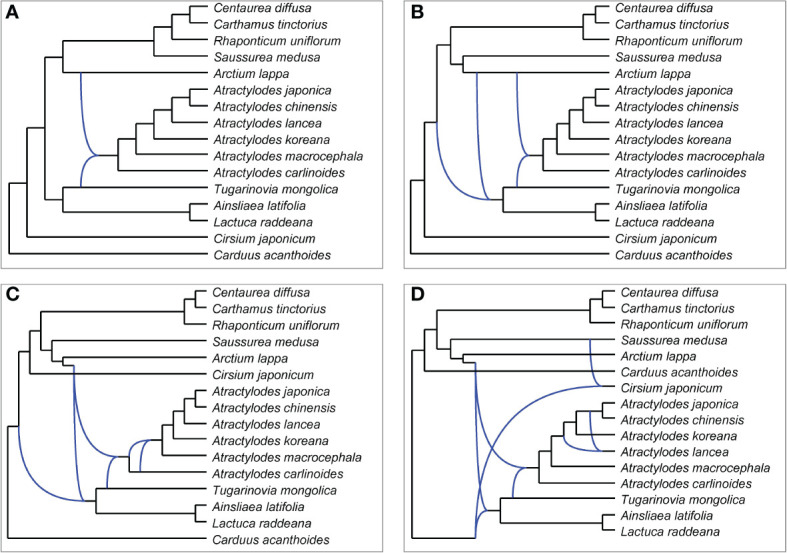
Networks representing plastid gene trees generated by PhyloNet MPL, allowing a maximum of 1 **(A)**, 2 **(B)**, 3 **(C)**, or 4 **(D)** reticulations.

PhyloNet was used to further assess putative hybridization events in the phylogeny. The analysis indicated that certain loci in the genome of *Atractylodes* shared a most recent common ancestor with loci in that of *Arctium lappa*, and others shared a most recent common ancestor with loci in that of *Tugarinovia mongolica*; this was the case in all networks allowing 1-4 reticulations (**Figure 7**). When 4 reticulations were allowed, the resulting network showed that *A. lancea* may have hybridized with the common ancestor of *A. chinensis* and *A. japonica*. The results of ABBA–BABA tests showed that *A. chinensis* was more closely related to *A. japonica* than to *A. lancea* (**Supplementary Table 12**).

## Discussion

Atractylodes is a small genus of the Asteraceae family mainly distributed across East Asia. It is a cross-pollination plant group, meaning that its morphological character is extremely susceptible to environmental factors (Hu et al., 2000). The phylogenetic relationships and the taxonomic treatment within the entire genus are intricate due to the continuous nature of variation in its morphological features (Peng et al., 2012). Atractylodes rhizomes distributed at low altitudes are bead-shaped and run horizontally; however, with changes in altitude and the ecological environment, rhizomes at medium altitudes appear in clumps and grow obliquely downwards (Zhu, 1981). The petioles and degree of leaf-splitting of the genus are still undergoing continuous evolution (Peng and Wang, 2007). In some instances, the lower and middle cauline leaves are petiolate, whereas basal leaves are sometimes subsessile (Hu et al., 2000). Although the leaf blades are generally divided into 3-5 pinnately arranged segments, they are occasionally undivided near the base, with a few small spiny lobes (Zhu, 1981). The 24 Atractylodes samples collected in this study also exhibited several continuously varying morphological features, especially those samples cultivated in Hubei province.

Consistent with previous studies, the phylogenetic trees presented here indicate that A. carlinoides is a basal species in the Atractylodes genus, and is a sister of the remainder of the Atractylodes species. Unlike one previous study predicting that A. macrocephala may be an A. chinensis and A. lancea hybrid (Wang et al., 2020), this study found that A. macrocephala forms an independent branch, with a bootstrap value of 100 in the case of both plastid data and nrDNA data. Regarding A. koreana, this species is mainly distributed in the Liaoning and Shandong provinces of China. Its lower and middle cauline leaves are undivided, which represents a point of distinction from the morphological characteristics of the other Atractylodes species. However, the ITS genotype of A. koreana was found here to be consistent with that of A. chinensis, and this also has been reported in a previous study (Shiba et al., 2006). A. chinensis and A. lancea are generally discriminated on the basis of differences in the shapes of their leaves. However, Shi has indicated that these morphological differences are unstable (Zhu, 1981), and past authors have been misled because the number of specimens they possessed was extremely limited, resulting in a poor understanding of the polytype of this species. Here, ten samples of A. lancea formed an independent clade in an analysis using nrDNA data from both wild and cultivated samples. A. japonica has long petioles, and the leaf blades generally divide almost to the base into 3-5 segments; these traits constitute obvious differences from other species in the A. lancea complex. This label has been considered a synonym for A. lancea, as recorded in the latest version of Flora of China. However, this study identified multiple strands of supporting evidence for a close relationship between A. japonica and other variations of the A. lancea complex in the nrDNA concatenated sequence-based phylogeny. Moreover, species trees obtained using PhyloNet confirmed that A. japonica is closely related to A. chinensis but separate from A. lancea.

Cytonuclear discordance can be caused by many factors, such as ancient hybridization and gene introgression. It is a common phenomenon in plant systematics and has been reported in many genera, such as section *Galoglychia* (Renoult et al., 2009) and *Cotoneaster* (Meng et al., 2021). Existing cpDNA and nuclear genetic evidence has revealed that sect. *Galoglychia* has many obvious nuclear–cytoplasmic phylogenetic tree conflicts, which are likely to be caused by ancient hybridization followed by gene introgression. In the case of *Cotoneaster*, sequences of the complete plastid genomes and 204 low-copy nuclear genes of 69 species were used for the phylogenetic analysis, and the results revealed there were conflicts between the plastid genome and low-copy nuclear phylogenies at both the species and clade levels. These instances of cytonuclear discordance may be caused by frequent hybridization events and incomplete lineage sorting (ILS). For species of *Atractylodes*, gene introgression usually results from natural hybridization among closely related species in sympatric populations (Hu et al., 2000). Shiba et al. have indicated that the continuous morphological variation in features between *A. lancea* and *A. chinensis* may be caused by the presence of such hybrids. Interspecific hybridization between *A. lancea* and *A. chinensis* has been observed in 25 samples containing nucleotide additives (Peng and Wang, 2007). Here, the phylogenetic network analysis of plastid genes revealed that *A. lancea* may have hybridized with the common ancestor of *A. chinensis* and *A. japonica.*

In conclusion, the results of this study tended to support treatment of *A. japonica* as an independent species. Although samples of *A. lancea* formed an independent clade, the other species in the *A. lancea* complex were still mingled with one another due to a complicated pattern of evolution in this genus, as shown by the phylogeny according to the plastid genomic data. In addition, this study revealed extensive discordance based on the cytonuclear genomic data, primarily involving the *A. lancea* complex. In future research, analysis of the A. lancea complex with sufficient single-copy nuclear genes or use of a reduced-representation genomic approach will be necessary to clarify the genetic differentiation.

## Data availability statement

The datasets presented in this study can be found in online repositories. The names of the repository/repositories and accession number(s) can be found in the article/**Supplementary Material**.

## Author contributions

LS, JL, and XiaoZ conceived and designed the study. HX, CZ, XinZ, QW, and YL collected and identified the plant materials. JL, MS, and HX performed the experiments. JL, MS, ZZ, and WK analyzed the data. JL, LS, MS, and HX wrote the manuscript. JL, LS, and XiaoZ revised the manuscript. All authors contributed to the article and approved the submitted version.

## Funding

This work was supported by National Key R&D Program: Intergovernmental Cooperation in International Science and Technology Innovation (2022YFE0119300), China Postdoctoral Science Foundation (2022M720504), Beijing Municipal Natural Science Foundation (7202136), the National Natural Science Foundation of China (81703659), Guangxi Science and Technology base and talent project (AD22080012).

## Acknowledgments

We thank Dr Ran Wei, State Key Laboratory of Systematic and Evolutionary Botany, Institute of Botany, Chinese Academy of Sciences, for providing help with the phylogenic analysis conducted in this study.

## Conflict of interest

The authors declare that the research was conducted in the absence of any commercial or financial relationships that could be construed as a potential conflict of interest.

## Publisher’s note

All claims expressed in this article are solely those of the authors and do not necessarily represent those of their affiliated organizations, or those of the publisher, the editors and the reviewers. Any product that may be evaluated in this article, or claim that may be made by its manufacturer, is not guaranteed or endorsed by the publisher.

## Abbreviations

IR, inverted repeat region; LSC, large single copy region; SSC, small single copy region; tRNA, transfer RNA; rRNA, ribosomal RNA; SSR, simple sequence repeats; ML, Maximum Likelihood.
