# Supplementary material for: Phylogenomic analyses based on the plastid genome and concatenated nrDNA sequence data reveal cytonuclear discordance in genus Atractylodes (Asteraceae: Carduoideae)
Source: Front Plant Sci. 2022 Dec 1;13:1045423. doi: 10.3389/fpls.2022.1045423 (PMC9752137; doi:10.3389/fpls.2022.1045423)
Supplement: Supplementary file 1 [file DataSheet_1.pdf]

Supplementary Materials

Supplementary Figures

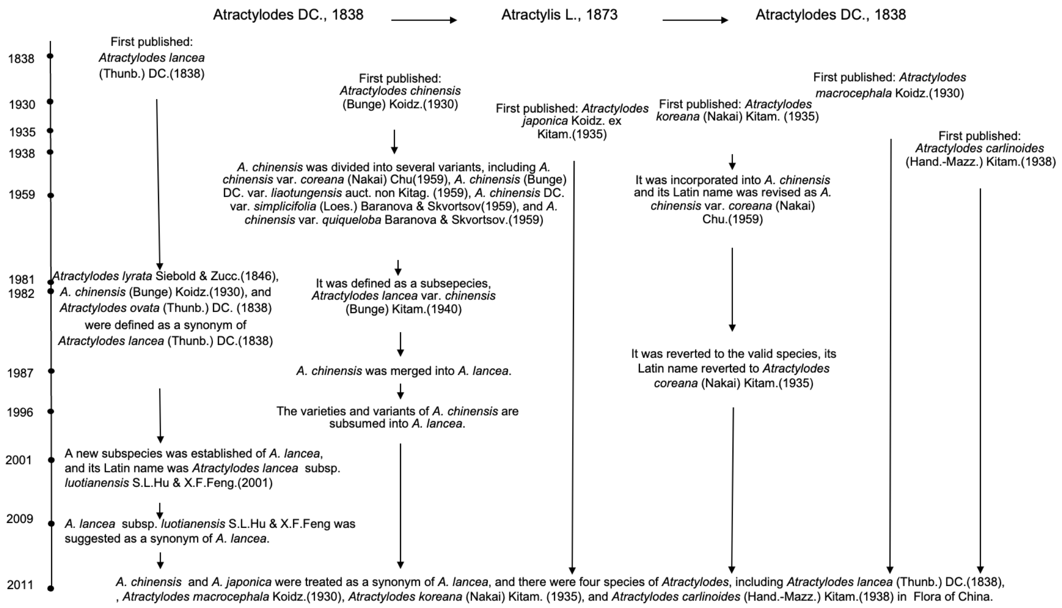

Supplementary Figure 1. The nomenclatural history of the *Atractylodes* species.

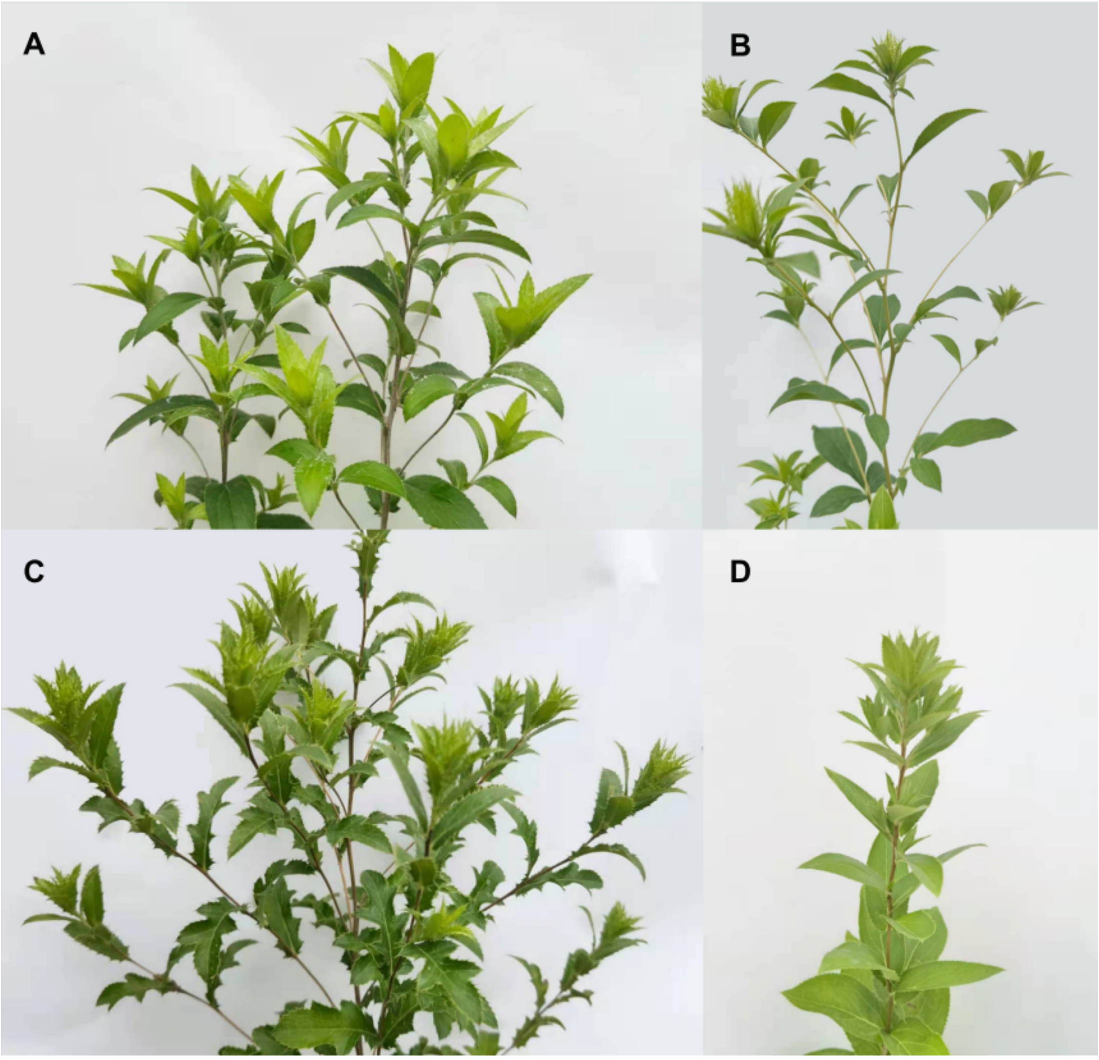

Supplementary Figure 2. The origin plants of the *Atractylodes lancea* complex species. A: *A. lancea*, B: *A. japonica*, C: *A. chinensis*, D: *A. koreana*.

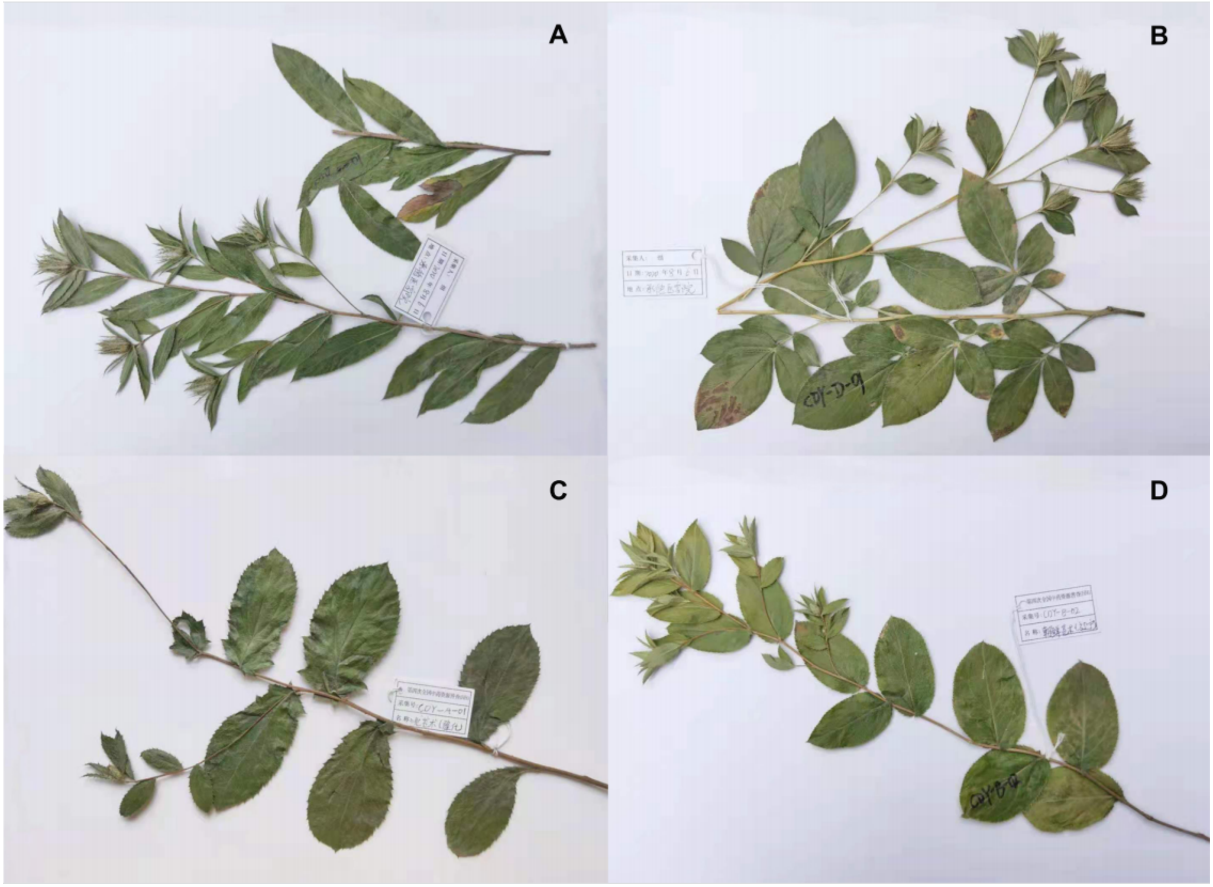

Supplementary Figure 3. The specimens of the *Atractylodes lancea* complex species. A: *A. lancea*, B: *A. japonica*, C: *A. chinensis*, D: *A. koreana.*

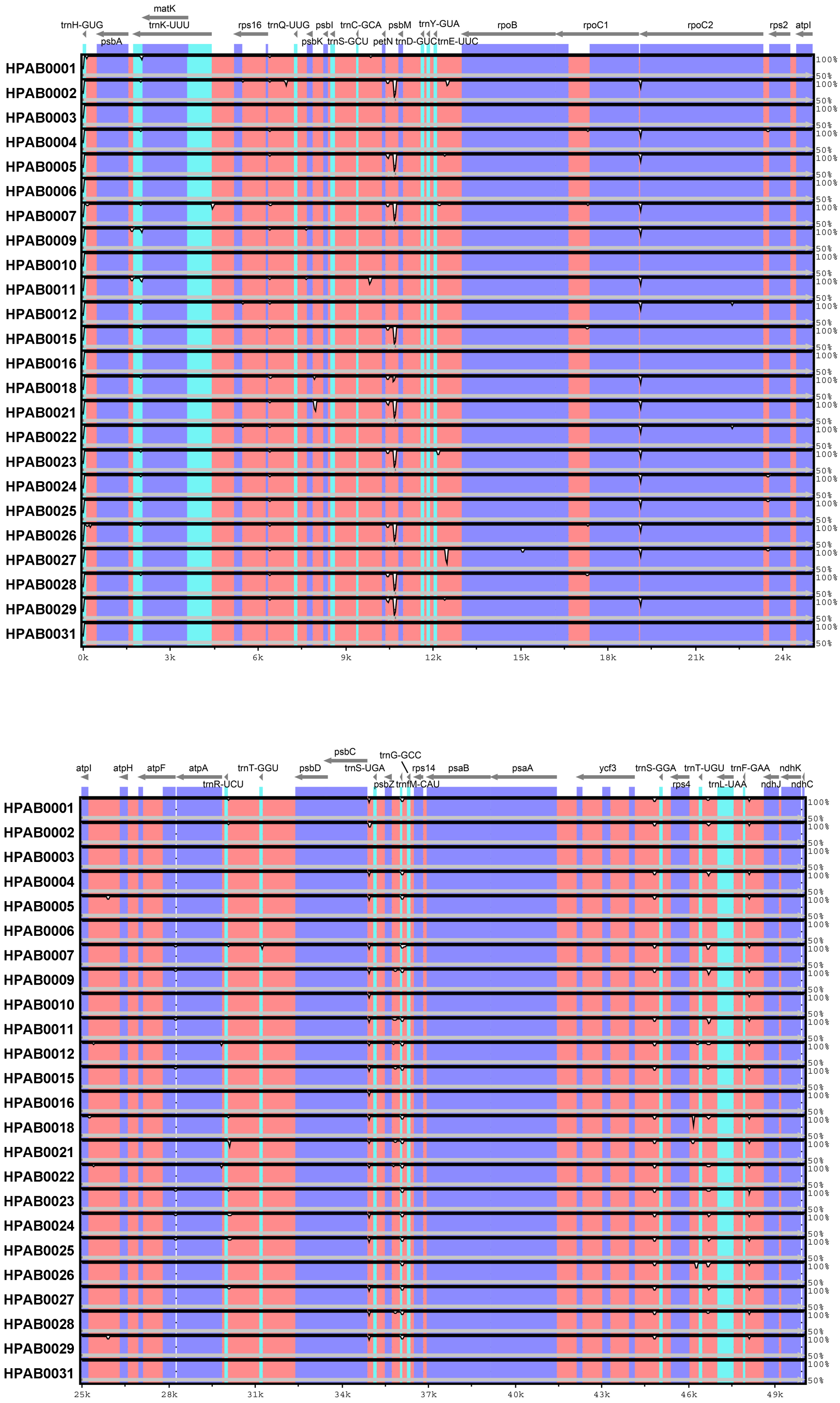
matK
trnH-GUG
trnK-UUU
psbA
rps16
trnQ-UUG
psbI
trnC-GCA
psbM
trnY-GUA
rpoB
rpoC1
rpoC2
rps2
atpI
psbK
trnS-GCU
petN
trnD-GUC
trnE-UUC
HPAB0001
HPAB0002
HPAB0003
HPAB0004
HPAB0005
HPAB0006
HPAB0007
HPAB0009
HPAB0010
HPAB0011
HPAB0012
HPAB0015
HPAB0016
HPAB0018
HPAB0021
HPAB0022
HPAB0023
HPAB0024
HPAB0025
HPAB0026
HPAB0027
HPAB0028
HPAB0029
HPAB0031
100%
50%
0k
3k
6k
9k
12k
15k
18k
21k
24k
psbC
trnG-GCC
atpI
atpH
atpF
atpA
trnT-GGU
psbD
trnS-UGA
rps14
psaB
psaA
ycf3
trnS-GGA
trnT-UGU
trnF-GAA
ndhK
trnR-UCU
psbZ
trnfM-CAU
rps4
trnL-UAA
ndhJ
ndhC
25k
28k
31k
34k
37k
40k
43k
46k
49k

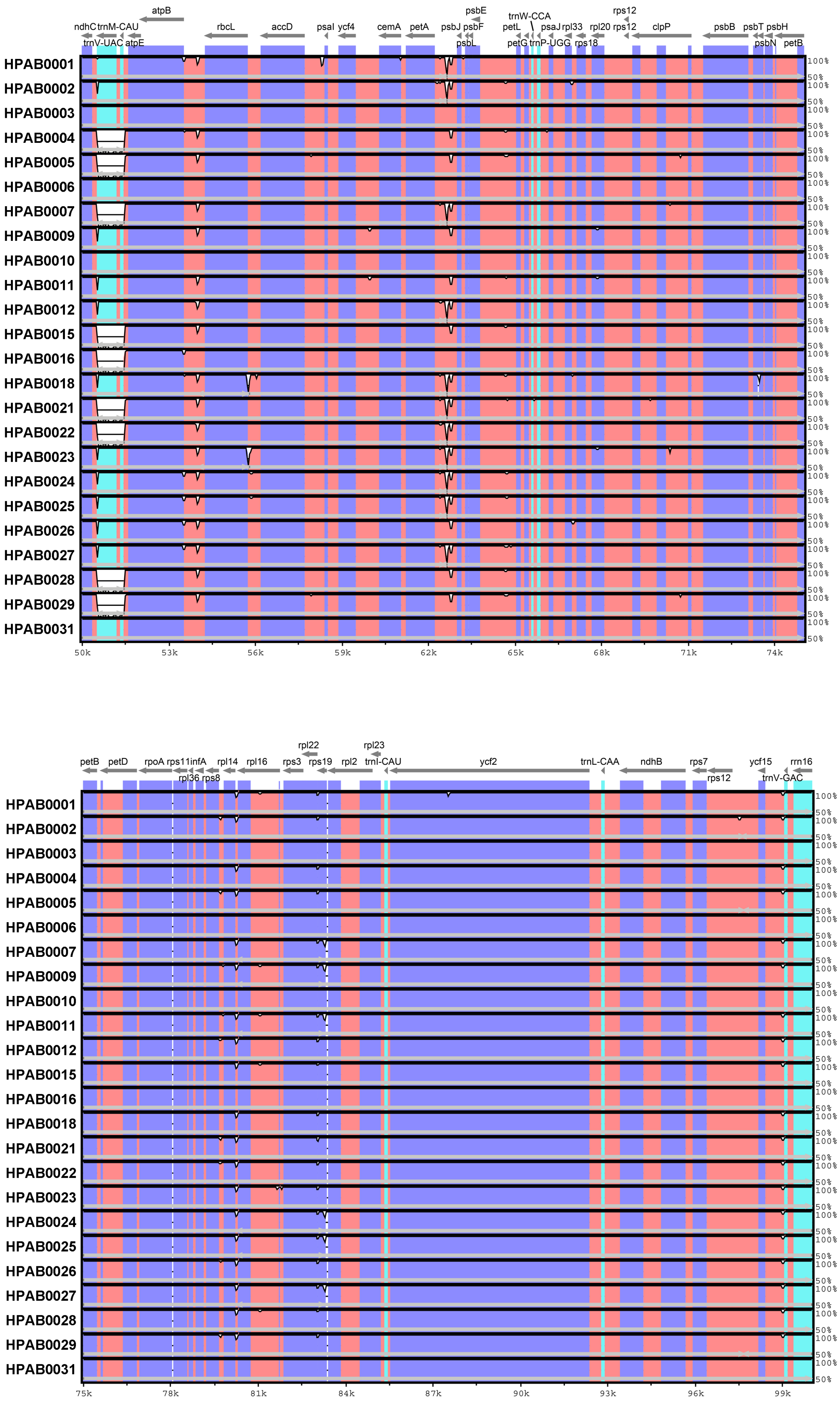

atpB
ndhC trnM-CAU
trnV-UAC atpE
rbcL
accD
psaI ycf4
cemA
petA
psbJ psbF psbE
psbL
petL petG trnW-CCA trnP-UGG
psaJ rpl33 rps18
rpl20 rps12
clpP
psbB
psbT psbN psbH
petB
HPAB0001
HPAB0002
HPAB0003
HPAB0004
HPAB0005
HPAB0006
HPAB0007
HPAB0009
HPAB0010
HPAB0011
HPAB0012
HPAB0015
HPAB0016
HPAB0018
HPAB0021
HPAB0022
HPAB0023
HPAB0024
HPAB0025
HPAB0026
HPAB0027
HPAB0028
HPAB0029
HPAB0031
100%
50%
50k 53k 56k 59k 62k 65k 68k 71k 74k
petB petD
rpoA rps11 infA rpl36 rps8
rpl14 rpl16
rps3 rpl22 rps19
rpl2 rpl23 trnI-CAU
ycf2
trnL-CAA
ndhB
rps7 rps12
ycf15 trnV-GAC
rrn16
75k 78k 81k 84k 87k 90k 93k 96k 99k

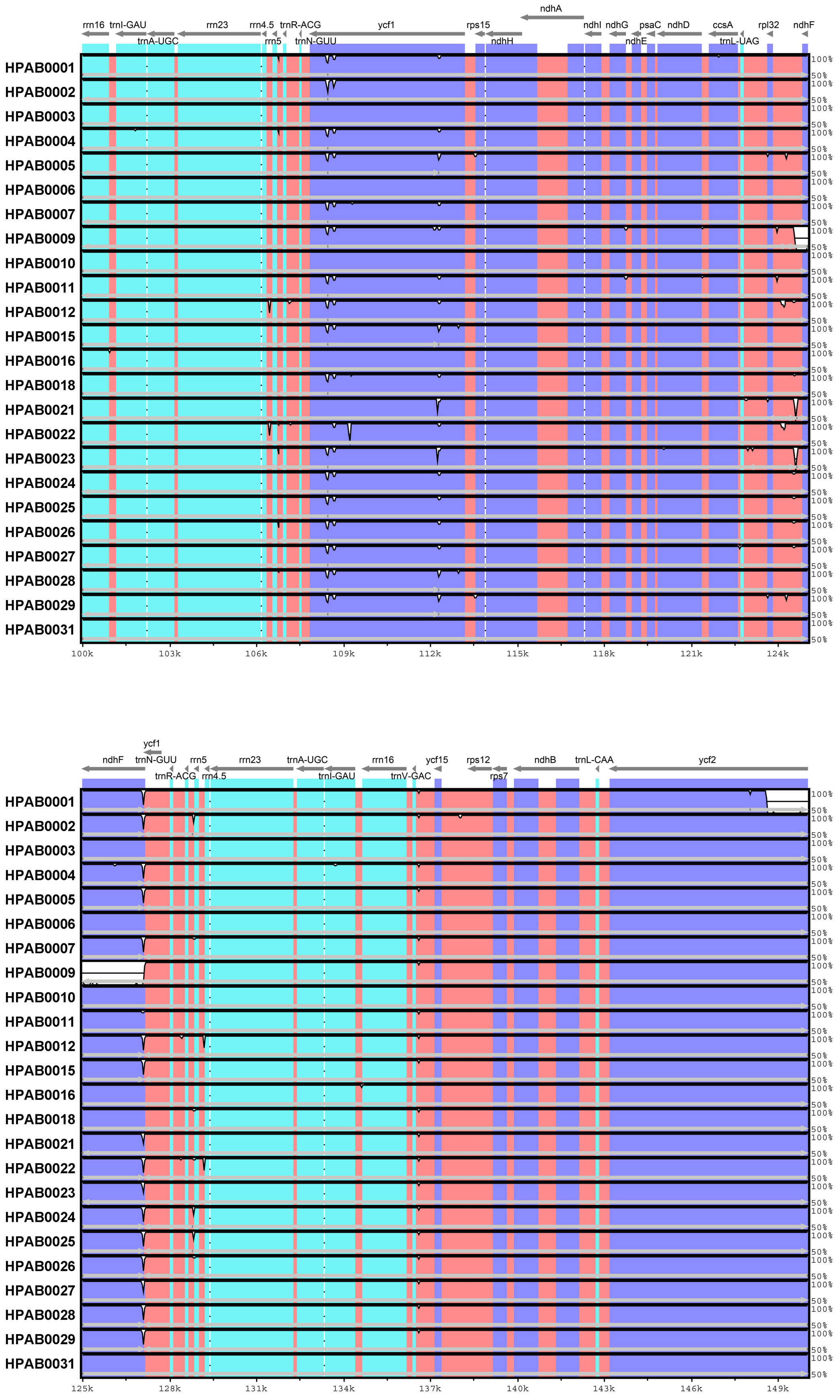

rrn16
trnI-GAU
trnA-UGC
rrn23
rrn4.5
rrn5
trnR-ACG
trnN-GUU
ycf1
rps15
ndhH
ndhA
ndhI
ndhG
ndhE
psaC
ndhD
ccsA
trnL-UAG
rpl32
ndhF
HPAB0001
HPAB0002
HPAB0003
HPAB0004
HPAB0005
HPAB0006
HPAB0007
HPAB0009
HPAB0010
HPAB0011
HPAB0012
HPAB0015
HPAB0016
HPAB0018
HPAB0021
HPAB0022
HPAB0023
HPAB0024
HPAB0025
HPAB0026
HPAB0027
HPAB0028
HPAB0029
HPAB0031
100%
50%
100k
103k
106k
109k
112k
115k
118k
121k
124k
ycf1
ndhF
trnN-GUU
trnR-ACG
rrn5
rrn4.5
rrn23
trnA-UGC
trnI-GAU
rrn16
trnV-GAC
ycf15
rps12
rps7
ndhB
trnL-CAA
ycf2
HPAB0001
HPAB0002
HPAB0003
HPAB0004
HPAB0005
HPAB0006
HPAB0007
HPAB0009
HPAB0010
HPAB0011
HPAB0012
HPAB0015
HPAB0016
HPAB0018
HPAB0021
HPAB0022
HPAB0023
HPAB0024
HPAB0025
HPAB0026
HPAB0027
HPAB0028
HPAB0029
HPAB0031
100%
50%
125k
128k
131k
134k
137k
140k
143k
146k
149k

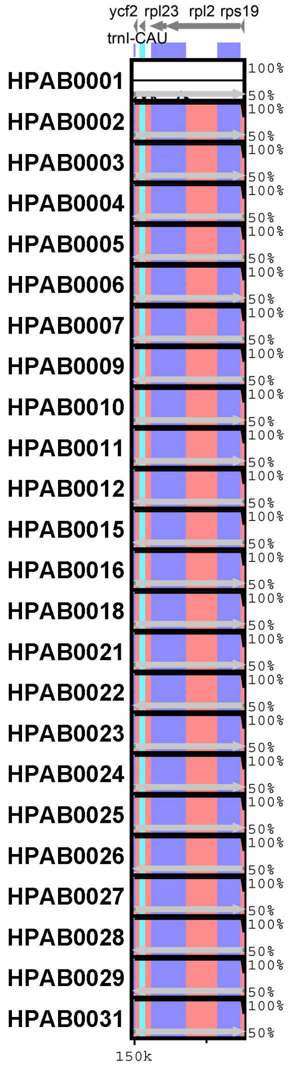

Supplementary Figure 4. A comparison between the 24 plastid genomes, with *A. chinensis* as a reference, using the mVISTA alignment program.

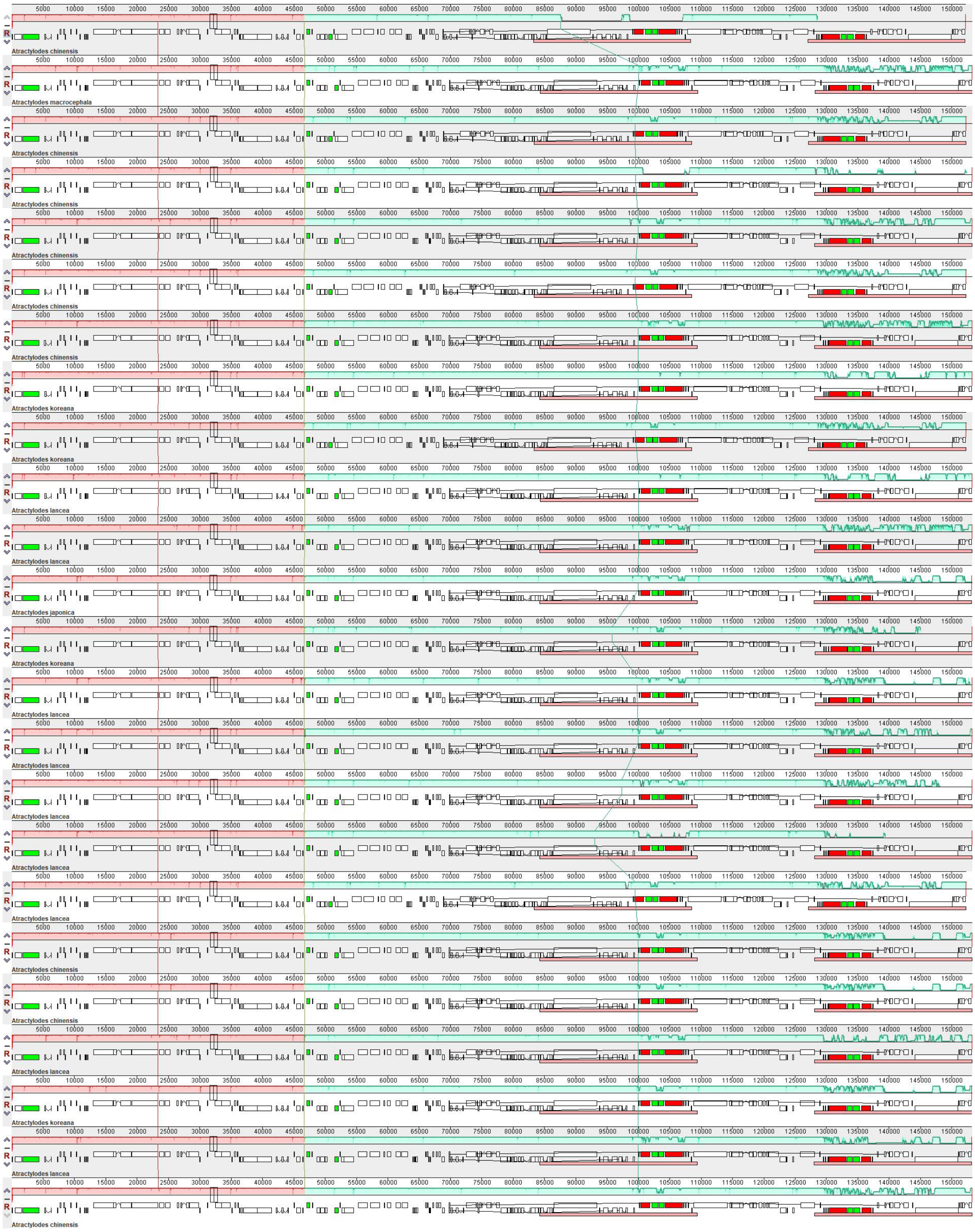

Supplementary Figure 5. A comparison between the whole plastid genomes of the *Atractylodes* species using the Mauve algorithm. The red LCBs indicate syntenic regions, while the histograms within each block represent the degree of sequence similarity. The rRNA, protein-coding, and tRNA gene annotations are denoted by the red, white, and green boxes, respectively.

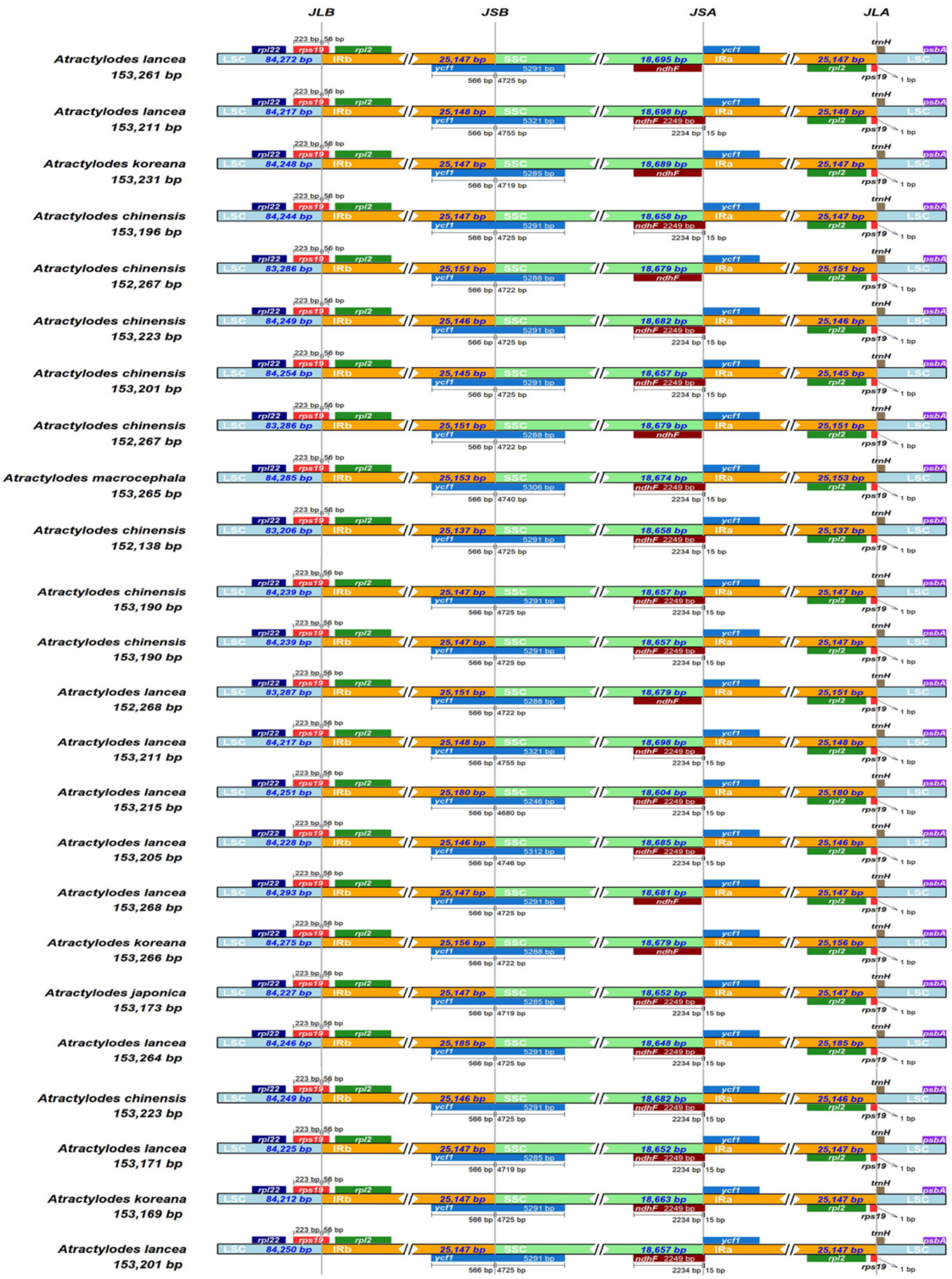

Supplementary Figure 6. A comparison of the LSC and IRb border region, as well as SSC and IRa border region for the five *Atractylodes* species. JLB: junction of LSC and IRb; JSA: junction of SSC and IRa.

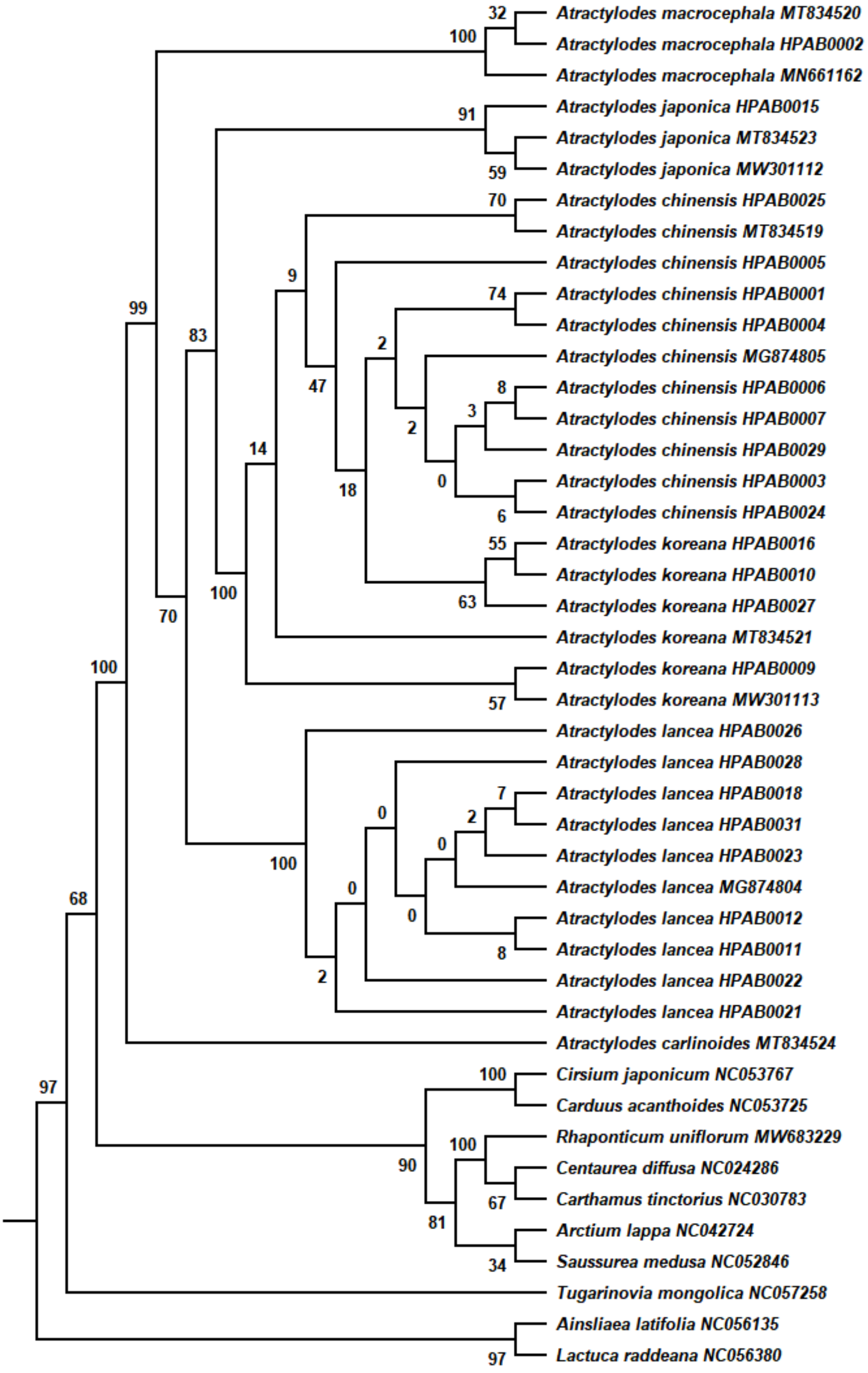

Supplementary Figure 7. The phylogenetic tree constructed using the ML based on the concatenated nrDNA sequences. The numbers above the branches represent the ML bootstrap values.

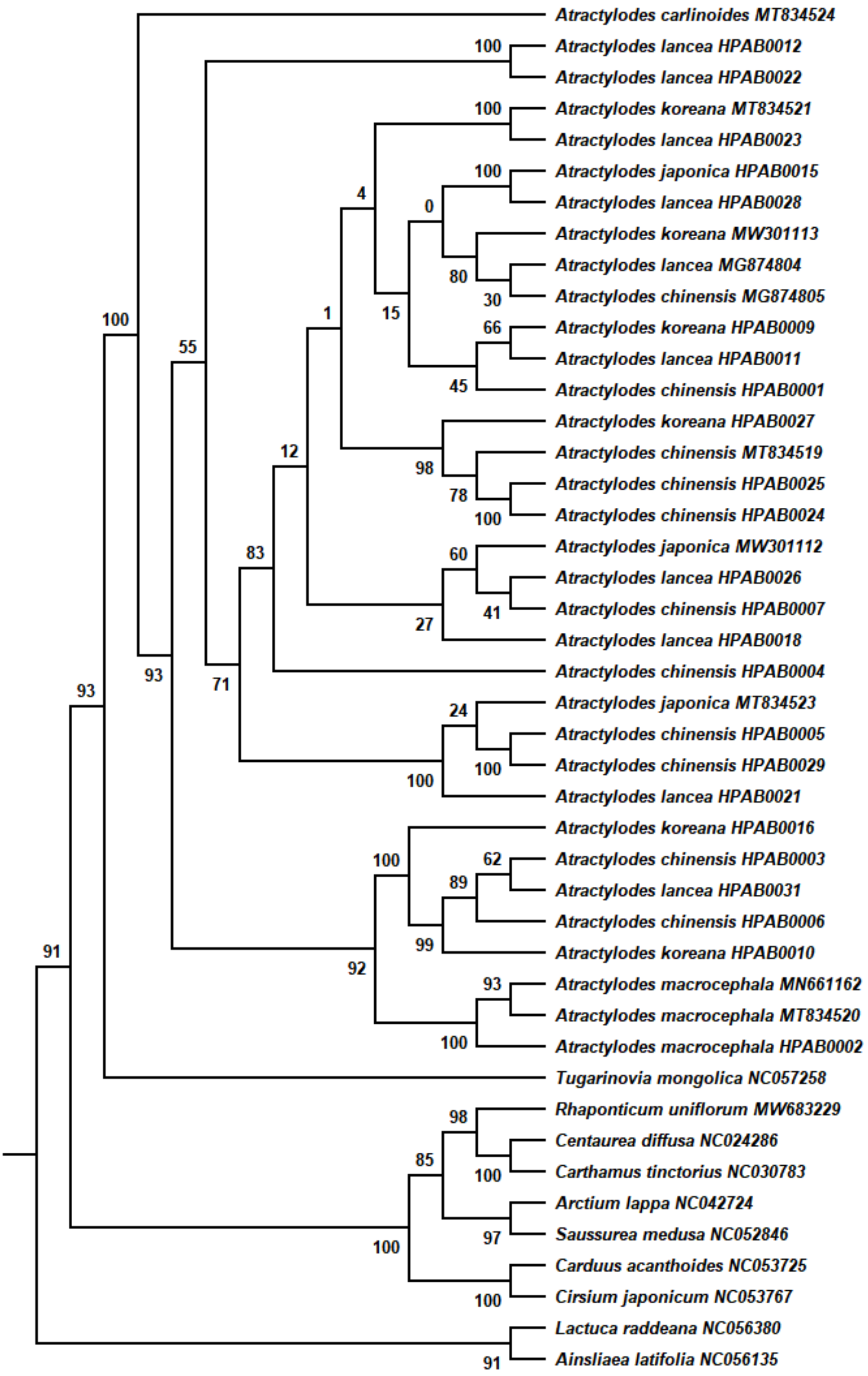

Supplementary Figure 8. The phylogenetic tree constructed using the ML based on the whole plastid genomes. The numbers above the branches represent the ML bootstrap values.

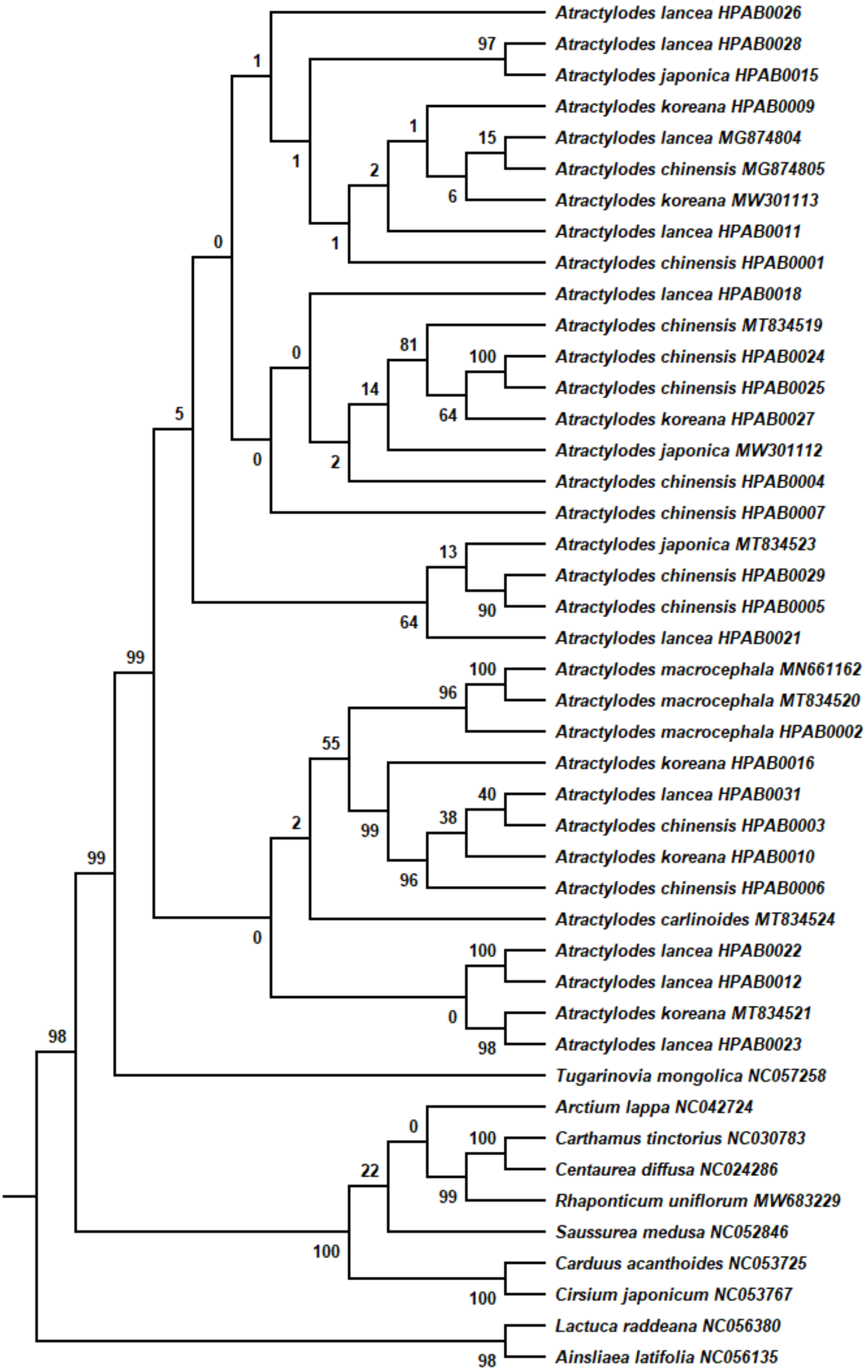

Supplementary Figure 9. The phylogenetic tree constructed using the ML based on 73 conserved plastid protein-coding genes. The numbers above the branches represent the ML bootstrap values.

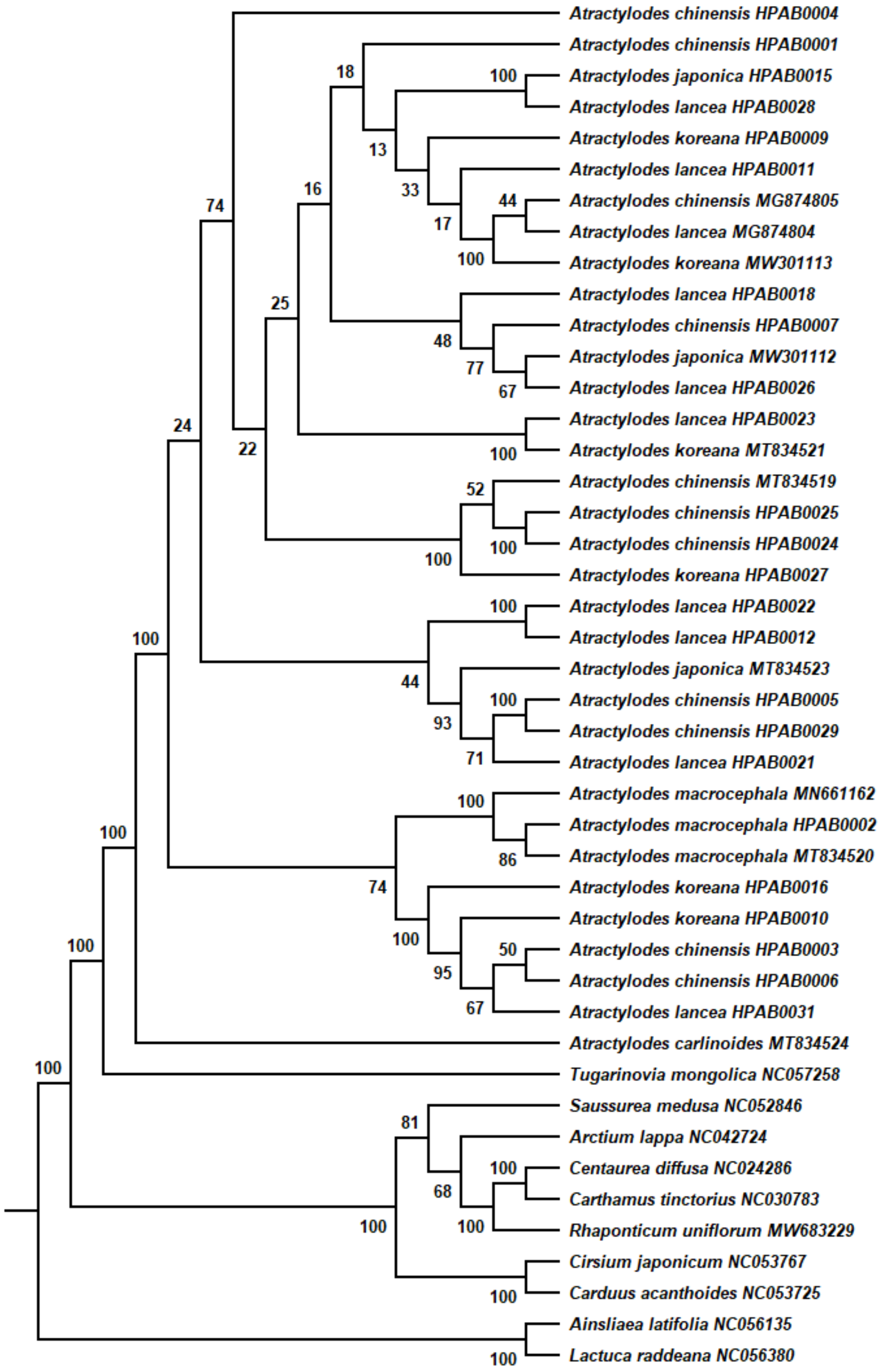

Supplementary Figure 10. The phylogenetic tree constructed using the ML based on 88 IGS regions. The numbers above the branches represent the ML bootstrap values.

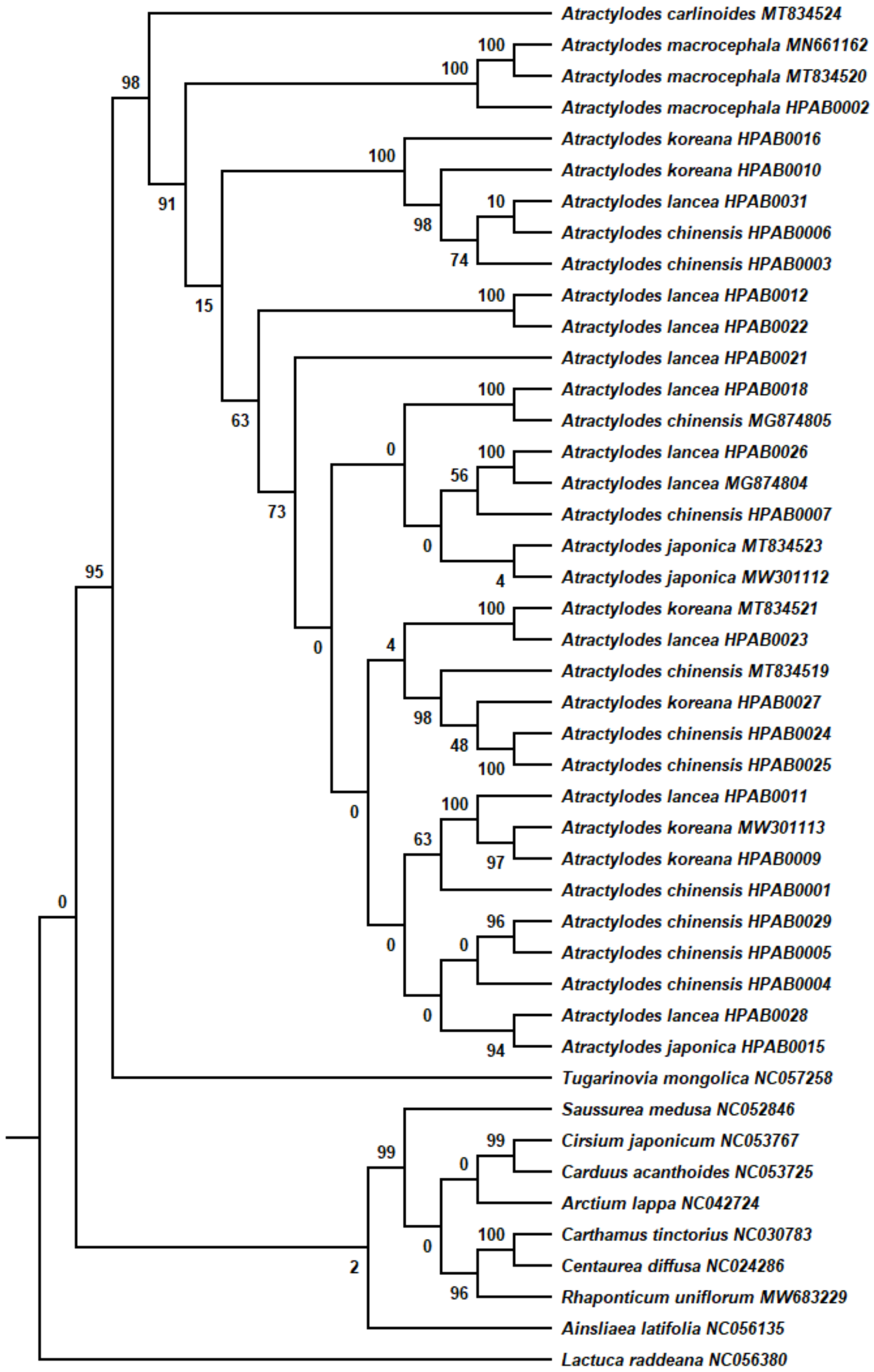

Supplementary Figure 11. The phylogenetic tree constructed using the ML based on 95 common genes. The numbers above the branches represent the ML bootstrap values.

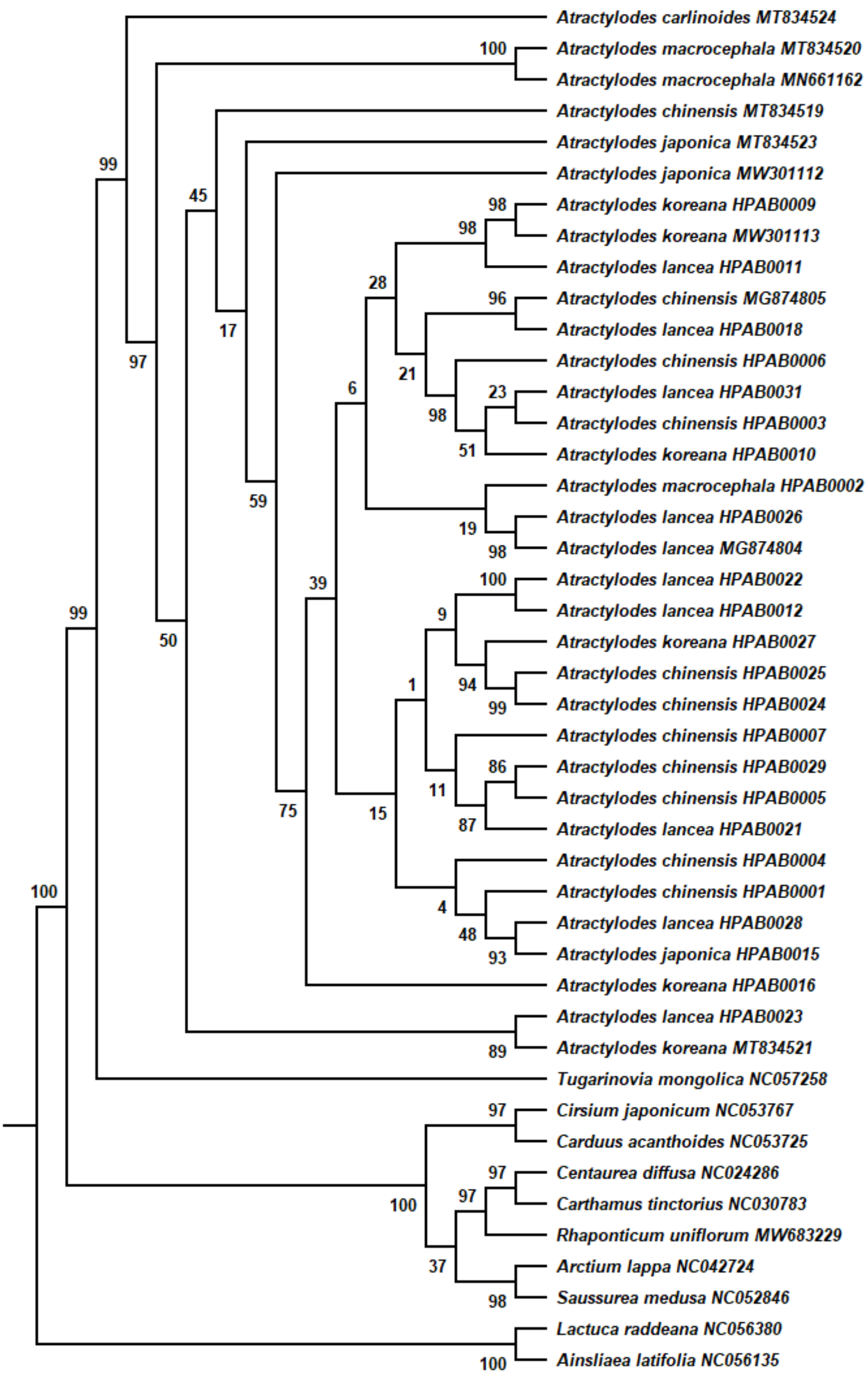

Supplementary Figure 12. The phylogenetic tree constructed using the ML based on 73 protein sequences. The numbers above the branches represent the ML bootstrap values.

Supplementary Tables

Supplementary Table 1. The samples information of *Atractylodes* species.

Source	Voucher specimen	Species	Collection locality	Identifier	Accession number （chloroplast genome）	Accession number （complete rDNA）
This study	HPAB0001	*A. chinensis*	Beijing Medicinal Botanical Garden	Qiuling Wang	MZ460962	MZ456956
	HPAB0002	*A. macrocephala*	Beijing Medicinal Botanical Garden	Qiuling Wang	MZ460963	MZ456957
	HPAB0003	*A. chinensis*	Weichang, Chengde, Hebei	Qiuling Wang	MZ460964	MZ456958
	HPAB0004	*A. chinensis*	Weichang, Chengde, Hebei	Qiuling Wang	MZ460965	MZ456959
	HPAB0005	*A. chinensis*	Weichang, Chengde, Hebei	Qiuling Wang	MZ460966	MZ456960
	HPAB0006	*A. chinensis*	Longhua, Chengde, Hebei	Chunying Zhao	MZ460967	MZ456961
	HPAB0007	*A. chinensis*	Longhua, Chengde, Hebei	Chunying Zhao	MZ460968	MZ456962
	HPAB0009	*A. koreana*	Shenyang, Liaoning	Chunying Zhao	MZ460969	MZ456963
	HPAB0010	*A. koreana*	Shenyang, Liaoning	Chunying Zhao	MZ460970	MZ456964
	HPAB0011	*A. lancea*	Mao shan, Jurong, Jiangsu	Chunying Zhao	MZ460971	MZ456965
	HPAB0012	*A. lancea*	Mao shan, Jurong, Jiangsu	Chunying Zhao	MZ460972	MZ456966
	HPAB0015	*A. japonica*	Shenyang, Liaoning	Chunying Zhao	MZ460975	MZ456969
	HPAB0016	*A. koreana*	Donggang, Dandong, Liaoning	Qiuling Wang	MZ460976	MZ456970
	HPAB0018	*A. lancea*	Yingshan, Huanggang, Hubei	Qiuling Wang	MZ460977	MZ456971
	HPAB0021	*A. lancea*	Yingshan, Huanggang, Hubei	Qiuling Wang	MZ460978	MZ456972
	HPAB0022	*A. lancea*	Yingshan, Huanggang, Hubei	Qiuling Wang	MZ460979	MZ456973
	HPAB0023	*A. lancea*	Yingshan, Huanggang, Hubei	Qiuling Wang	MZ460980	MZ456974
	HPAB0024	*A. chinensis*	Beijing Medicinal Botanical Garden	Qiuling Wang	MZ460982	MZ456975
	HPAB0025	*A. chinensis*	Beijing Medicinal Botanical Garden	Yulin Lin	MZ460983	MZ456976
	HPAB0026	*A. lancea*	Mao shan, Jurong, Jiangsu	Yulin Lin	MZ460984	MZ456977

HPAB0027	*A. koreana*	Donggang, Dandong, Liaoning	Qiuling Wang	MZ460985	MZ456978
HPAB0028	*A. lancea*	Yingshan, Huanggang, Hubei	Qiuling Wang	MZ460986	MZ456979
HPAB0029	*A. chinensis*	Meixian, Baoji, Shanxi	Qiuling Wang	MZ460987	MZ456980
HPAB0031	*A. lancea*	Tiantaishan, Hongan, Hubei	Xinlei Zhao	MZ460981	MZ456981

Supplementary Table 2. The plastid genome accession number and SRA number for data downloaded from GenBank

Source	Genus	Species	Accession number（chloroplast genome）	Sequence Read Archive (SRA)
Genbank	*Atractylodes*	*A. lancea*	MG874804	SRR10598214
		A. chinensis	MG874805	SRR10725998
		A. macrocephala	MN661162	SRR10597911
		A. chinensis	MT834519	SRR13449941
		A. macrocephala	MT834520	SRR13449940
		A. koreana	MT834521	SRR13449939
		A. japonica	MT834523	SRR13449937
		A. carlinoides	MT834524	SRR13449936
		A. japonica	MW301112	SRR13181860
		A. koreana	MW301113	SRR13188655
	Arctium	*Arctium lappa*	NC042724	SRR9845549
	Carduus	*Carduus acanthoides*	NC053725	SRR12199770
	Carthamus	*Carthamus tinctorius*	NC030783	SRR3623470
	Centaurea	*Centaurea diffusa*	NC024286	SRR2729212
	Cirsium	*Cirsium japonicum*	NC053767	SRR12975765

Rhaponticum	*Rhaponticum uniflorum*	MW683229	SRR14270266
Saussurea	*Saussurea medusa*	NC052846	SRR14470736
Tugarinovia	*Tugarinovia mongolica*	NC057258	ERR4180103
Ainsliaea (outgroup)	*Ainsliaea atifolia*	NC056135	SRR16072379
Lactuca (outgroup)	*Lactuca raddeana*	NC056380	SRR13311444

Supplementary Table 3. The plastid genome features of *Atractylodes species*.

Features	Size(bp)	Size of LSC(bp)	Size of SSC(bp)	Size of IR(bp)	GC content(%)	GC content of LSC(%)	GC content of SSC(%)	GC content of IR(%)	Unique genes	Protein coding genes	tRNA genes	rRNA genes
A. chinensis	152,267~ 153,223	83,206~ 84,254	18,657~ 18,682	25,137~ 25,151	37.69~37.77	35.80~35.90	31.50~31.57	43.16~43.17	113	80	29	4
A. koreana	152,283~ 153,266	83,302~ 84,275	18,663~ 18,689	25,147~ 25,156	37.69~37.75	35.80~37.70	31.51~31.54	43.15~43.17	113	80	29	4
A. lancea	152,268~ 153,268	83,287~ 84,293	18,604~ 18,698	25,146~ 25,185	37.69~37.75	35.80~37.70	31.49~31.56	43.14~43.17	113	80	29	4
A. japonica	153,173	84,227	18,652	25,147	37.71	35.81	31.54	43.17	113	80	29	4
A. macrocephala	153,265	84,285	18,674	25,153	37.7	35.80	31.56	43.16	113	80	29	4

Supplementary Table 4. The Gene category of *Atractylodes* plastid genome.

Category	Group	Genes
Photosynthesis	Subunits of photosystem I	*psaA, psaB, psaI, psaJ, psaC*
	Subunits of photosystem II	*psbA, psbB, psbC, psbD, psbE, psbF, psbH, psbI, psbJ, psbK, psbL, psbM, psbN, psbT, psbZ*
	Subunits of NADH dehydrogenase	*ndhA*, ndhB*, ndhC, ndhD, ndhE, ndhF, ndhG, ndhH, ndhI, ndhJ, ndhK*
	Subunits of cytochrome b/f complex	*petA, petB*, petD*, petL, petN, petG*
	Subunits of ATP synthase	*atpA, atpB, atpE, atpF*, atpH, atpI*
	Large subunit of Rubisco	*rbcL*
Protein synthesis and self-replication	Large subunits of ribosome	*rpl14, rpl16*, rpl2*, rpl20, rpl22, rpl23, rpl32, rpl33, rpl36*
	Small subunits of ribosome	*rps2, rps3, rps4, rps7, rps8, rps11, rps12**, rps14, rps15, rps16*, rps18, rps19*
	DNA dependent RNA polymerase	*rpoA, rpoB, rpoC1*, rpoC2*
	Ribosomal RNAs	*rrn5, rrn4.5, rrn23, rrn16*
	Transfer RNAs	*trnA-UGC*, trnC-GCA, trnD-GUC, trnE-UUC, trnF-GAA, trnG-GCC, trnH-GUG, trnI-CAU, trnI-GAU*, trnK-UUU*, trnL-CAA, trnL-UAA*, trnL-UAG, trnM-CAU, trnN-GUU, trnP-UGG, trnQ-UUG, trnR-ACG, trnR-UCU, trnS-GCU, trnS-GGA, trnS-UGA, trnT-GGU, trnT-UGU, trnV-GAC, trnV-UAC*, trnW-CCA, trnY-GUA, trnfM-CAU*
Other genes	Maturase	*matK*
	Protease	*clpP***
	Envelope membrane protein	*cemA*
	Acetyl-CoA carboxylase	*accD*
	C-type cytochrome synthesis gene	*ccsA*

Unknown function	Translational initiation factor	*infA*
	hypothetical chloroplast reading frames (ycf)	*ycf1, ycf15, ycf2, ycf3**, ycf4*

Note: "*" indicated that the gene contains one intron; "**" indicated that the gene contains two introns.

Supplementary Table 5. The concatenated nrDNA sequence features of *Atractylodes* species.

Features	whole length	18S length	ITS1 length	5.8S length	ITS2 length	28S length	GC content	GC content of 18S	GC content of ITS1	GC content of 5.8S	GC content of ITS2	GC content of 28S
A. chinensis	5,849	1,809	259	158	229	3,394	55.48~55.51	49.47	62.93~63.71	54.43	62.93~63.71	57.28~57.31
A. koreana	5,849	1,809	259	158	229	3,394	55.48~55.53	49.47	62.55~63.32	54.43	62.55~63.32	57.28~57.31
A. lancea	5,849	1,809	259	158	229	3,394	55.55	49.47	63.71	54.43	63.71	57.31
A. japonica	5,849	1,809	259	158	229	3,394	55.45	49.47	62.16	54.43	62.16	57.25
A. macrocephala	5,849	1,809	259	158	229	3,394	55.62	49.53	64.48	54.43	64.48	57.31

Supplementary Table 6. The relative synonymous codon usage (RSCU) in the *Atractylodes* plastid genomes.

Amino acid	Codon	***A. lancea***		***A. chinensis***		***A. koreana***		***A. japonica***		***A. macrocephala***	
		**Number**	**RSCU**	**Number**	**RSCU**	**Number**	**RSCU**	**Number**	**RSCU**	**Number**	**RSCU**
Ala	GCT	349	1.55	351	1.55	371	1.60	353	1.56	352	1.56
	GCC	210	0.93	211	0.93	208	0.90	212	0.93	210	0.93
	GCA	224	0.99	224	0.99	227	0.99	224	0.99	224	0.99

	GCG	119	0.53	117	0.52	120	0.52	118	0.52	117	0.52
Cys	TGT	277	1.14	276	1.15	267	1.14	277	1.15	275	1.15
	TGC	207	0.86	203	0.85	202	0.86	203	0.85	202	0.85
Asp	GAT	499	1.49	492	1.49	531	1.51	494	1.49	490	1.48
	GAC	169	0.51	169	0.51	170	0.49	171	0.51	171	0.52
Glu	GAA	671	1.46	669	1.45	698	1.46	671	1.45	668	1.45
	GAG	250	0.54	253	0.55	259	0.54	255	0.55	256	0.55
Phe	TTT	884	1.20	871	1.19	877	1.21	871	1.19	871	1.19
	TTC	589	0.80	592	0.81	572	0.79	590	0.81	590	0.81
Gly	GGT	402	1.31	399	1.31	408	1.31	401	1.31	400	1.31
	GGC	187	0.61	186	0.61	186	0.60	187	0.61	187	0.61
	GGA	402	1.31	397	1.31	419	1.34	402	1.31	396	1.30
	GGG	237	0.77	234	0.77	233	0.75	239	0.78	234	0.77
His	CAT	353	1.38	349	1.36	365	1.41	351	1.37	348	1.36
	CAC	159	0.62	161	0.64	153	0.60	162	0.63	162	0.64
Ile	ATT	807	1.20	806	1.17	820	1.22	811	1.17	806	1.17
	ATC	511	0.76	520	0.76	495	0.74	523	0.76	522	0.76
	ATA	699	1.03	733	1.07	701	1.04	738	1.07	736	1.07
Lys	AAA	896	1.35	897	1.33	896	1.36	905	1.33	897	1.33
	AAG	435	0.65	449	0.67	428	0.65	455	0.67	456	0.67
Leu	TTA	542	1.44	549	1.42	559	1.47	552	1.42	547	1.41
	TTG	542	1.43	557	1.44	555	1.47	559	1.44	560	1.44
	CTT	436	1.15	442	1.14	441	1.16	445	1.14	442	1.14
	CTC	221	0.58	227	0.59	215	0.57	228	0.59	227	0.58
	CTA	323	0.85	336	0.87	309	0.81	338	0.87	339	0.87
	CTG	204	0.54	212	0.55	202	0.53	213	0.55	216	0.56

Met	ATG	566	1.00	588	1.00	575	1.00	590	1	588	1.00
Asn	AAT	724	1.37	710	1.36	763	1.40	710	1.36	707	1.36
	AAC	334	0.63	336	0.64	324	0.60	336	0.64	335	0.64
Pro	CCT	272	1.17	273	1.17	283	1.20	275	1.17	276	1.18
	CCC	188	0.81	189	0.81	190	0.81	192	0.82	188	0.81
	CCA	329	1.42	328	1.41	328	1.40	334	1.42	328	1.41
	CCG	138	0.60	142	0.61	138	0.59	141	0.6	141	0.60
Gln	CAA	473	1.42	471	1.40	496	1.44	475	1.4	471	1.40
	CAG	195	0.58	201	0.60	192	0.56	203	0.6	202	0.60
Arg	AGA	402	1.87	387	1.83	399	1.87	389	1.83	384	1.83
	AGG	241	1.12	235	1.11	229	1.07	236	1.11	233	1.11
	CGT	186	0.87	188	0.89	196	0.92	192	0.9	188	0.89
	CGC	107	0.50	107	0.51	109	0.51	107	0.5	107	0.51
	CGA	221	1.03	220	1.04	224	1.05	222	1.04	219	1.04
	CGG	130	0.61	132	0.62	128	0.60	132	0.62	131	0.62
Ser	AGT	318	1.06	312	1.06	331	1.09	310	1.05	310	1.06
	AGC	243	0.80	234	0.79	241	0.80	232	0.79	232	0.79
	TCT	413	1.37	406	1.38	423	1.40	410	1.39	406	1.38
	TCC	356	1.18	352	1.20	356	1.18	352	1.19	351	1.19
	TCA	293	0.97	284	0.97	278	0.92	286	0.97	286	0.97
	TCG	186	0.62	178	0.61	187	0.62	178	0.6	178	0.61
Thr	ACT	357	1.28	353	1.28	374	1.32	355	1.28	353	1.28
	ACC	286	1.02	281	1.02	287	1.01	283	1.02	280	1.02
	ACA	290	1.04	287	1.04	291	1.03	289	1.04	286	1.04
	ACG	184	0.66	183	0.66	184	0.65	183	0.66	184	0.67
Val	GTT	388	1.36	389	1.35	394	1.38	393	1.34	390	1.35

	GTC	181	0.64	181	0.63	175	0.61	186	0.64	181	0.63
	GTA	409	1.44	419	1.45	411	1.44	425	1.45	420	1.45
	GTG	161	0.57	164	0.57	162	0.57	166	0.57	164	0.57
Trp	TGG	381	1.00	382	1.00	375	1.00	381	1	382	1.00
Tyr	TAT	585	1.32	564	1.31	601	1.35	565	1.31	561	1.31
	TAC	304	0.68	298	0.69	291	0.66	297	0.69	297	0.69
Stop	TAA	443	1.22	439	1.21	387	1.29	441	1.22	438	1.21
	TAG	308	0.84	313	0.87	263	0.84	314	0.87	313	0.87
	TGA	343	0.94	334	0.92	280	0.88	333	0.92	333	0.92
Total		22865	64.01	22864	64.02	22868	64.01	22861	64.03	22868	63.98

Supplementary Table 7. The plastid simple sequence repeats (cpSSRs) of the *Atractylodes* species. p: perfect microsatellites; c: compound. The structures of the microsatellite repeats are presented as repeat units in parenthesis and the numbers of the repeat units.

Sample ID	SSR nr.	SSR type	SSR	Size (bp)	Start (bp)	End (bp)	Region
A. macrocephala	1	p1	(T)11	11	1975	1985	LSC
	2	p1	(T)12	12	12926	12937	LSC
	3	p1	(A)10	10	13620	13629	LSC
	4	p1	(A)10	10	18675	18684	LSC
	5	p1	(T)10	10	23517	23526	LSC
	6	p1	(T)10	10	25242	25251	LSC
	7	p1	(A)10	10	25395	25404	LSC
	8	p1	(T)10	10	26336	26345	LSC
	9	p1	(T)11	11	28275	28285	LSC
	10	p1	(T)15	15	34956	34970	LSC
	11	p1	(A)10	10	35827	35836	LSC
	12	p1	(A)10	10	44241	44250	LSC
	13	p1	(T)11	11	46328	46338	LSC
	14	c	(T)11cattttatttttaattaaaaaataaaatg(A)14	55	46688	46742	LSC
	15	p1	(T)13	13	50516	50528	LSC
	16	p1	(T)11	11	50642	50652	LSC
	17	p1	(T)15	15	51499	51513	LSC
	18	p1	(T)11	11	54911	54921	LSC
	19	p1	(T)11	11	59542	59552	LSC
	20	p1	(T)18	18	63422	63439	LSC

	21	p1	(A)15	15	65668	65682	LSC
	22	p1	(T)11	11	71635	71645	LSC
	23	p1	(A)10	10	78005	78014	LSC
	24	p1	(T)10	10	78246	78255	LSC
	25	p1	(T)17	17	80684	80700	LSC
	26	p1	(A)14	14	107727	107740	IRB
	27	p1	(A)10	10	109877	109886	SSC
	28	p1	(A)10	10	110331	110340	SSC
	29	p1	(T)10	10	125565	125574	SSC
	30	p1	(T)14	14	129811	129824	IRA
A. chinensis	1	p1	(T)14	14	1974	1987	LSC
	2	p1	(C)10	10	5451	5460	LSC
	3	p1	(T)11	11	12924	12934	LSC
	4	p1	(A)10	10	13617	13626	LSC
	5	p1	(A)10	10	18672	18681	LSC
	6	p1	(T)10	10	23496	23505	LSC
	7	p1	(T)10	10	25221	25230	LSC
	8	p1	(A)10	10	25374	25383	LSC
	9	p1	(T)10	10	26315	26324	LSC
	10	p1	(T)12	12	28254	28265	LSC
	11	p1	(A)11	11	30066	30076	LSC
	12	p1	(T)15	15	34938	34952	LSC
	13	p1	(A)10	10	35801	35810	LSC
	14	p1	(A)10	10	44215	44224	LSC
	15	p1	(T)11	11	46297	46307	LSC
	16	c	(T)13cattttattttttaattaaaaaataaaatg(A)11	54	46657	46710	LSC

	17	p1	(T)16	16	50489	50504	LSC
	18	p1	(T)11	11	53902	53912	LSC
	19	p1	(T)11	11	58534	58544	LSC
	20	p1	(T)17	17	62414	62430	LSC
	21	p1	(A)13	13	64675	64687	LSC
	22	p1	(T)11	11	70633	70643	LSC
	23	p1	(A)10	10	77003	77012	LSC
	24	p1	(T)10	10	77244	77253	LSC
	25	p1	(T)13	13	79682	79694	LSC
	26	p1	(A)13	13	106727	106739	IRB
	27	p1	(A)10	10	108858	108867	SSC
	28	p1	(A)10	10	109312	109321	SSC
	29	p1	(T)10	10	124546	124555	SSC
	30	p1	(T)13	13	128815	128827	IRA
A. koreana	1	p1	(T)10	10	1981	1990	LSC
	2	p1	(T)11	11	12934	12944	LSC
	3	p1	(A)10	10	13627	13636	LSC
	4	p1	(T)12	12	17310	17321	LSC
	5	p1	(A)10	10	18681	18690	LSC
	6	p1	(T)10	10	23523	23532	LSC
	7	p1	(T)10	10	25248	25257	LSC
	8	p1	(A)10	10	25401	25410	LSC
	9	p1	(T)10	10	26342	26351	LSC
	10	p1	(T)10	10	28281	28290	LSC
	11	p1	(A)10	10	30091	30100	LSC
	12	p1	(T)14	14	34962	34975	LSC

	13	p1	(A)11	11	35882	35892	LSC
	14	p1	(A)10	10	44243	44252	LSC
	15	p1	(T)11	11	46330	46340	LSC
	16	p1	(A)11	11	46722	46732	LSC
	17	p1	(T)18	18	50505	50522	LSC
	18	p1	(T)10	10	51492	51501	LSC
	19	p1	(T)10	10	54899	54908	LSC
	20	p1	(T)10	10	59514	59523	LSC
	21	p1	(T)17	17	63399	63415	LSC
	22	p1	(A)14	14	65644	65657	LSC
	23	p1	(T)10	10	71604	71613	LSC
	24	p1	(A)10	10	77973	77982	LSC
	25	p1	(T)10	10	78214	78223	LSC
	26	p1	(T)13	13	80652	80664	LSC
	27	p1	(A)14	14	107684	107697	IRB
	28	p1	(A)10	10	109825	109834	SSC
	29	p1	(A)10	10	110279	110288	SSC
	30	p1	(T)14	14	129783	129796	IRA
A. lancea	1	p1	(T)11	11	12956	12966	LSC
	2	p1	(A)10	10	13649	13658	LSC
	3	p1	(T)12	12	17332	17343	LSC
	4	p1	(A)10	10	18703	18712	LSC
	5	p1	(T)11	11	23545	23555	LSC
	6	p1	(T)10	10	25271	25280	LSC
	7	p1	(A)10	10	25424	25433	LSC
	8	p1	(T)10	10	26365	26374	LSC

	9	p1	(T)10	10	28304	28313	LSC
	10	p1	(A)10	10	30114	30123	LSC
	11	p1	(T)14	14	34985	34998	LSC
	12	p1	(A)11	11	35904	35914	LSC
	13	p1	(A)10	10	44265	44274	LSC
	14	p1	(T)11	11	46352	46362	LSC
	15	p1	(A)13	13	46744	46756	LSC
	16	p1	(T)18	18	50529	50546	LSC
	17	p1	(T)10	10	51516	51525	LSC
	18	p1	(T)10	10	54923	54932	LSC
	19	p1	(T)10	10	59538	59547	LSC
	20	p1	(T)17	17	63423	63439	LSC
	21	p1	(A)14	14	65668	65681	LSC
	22	p1	(T)10	10	71628	71637	LSC
	23	p1	(A)10	10	77997	78006	LSC
	24	p1	(T)10	10	78238	78247	LSC
	25	p1	(T)13	13	80676	80688	LSC
	26	p1	(A)14	14	107708	107721	IRB
	27	p1	(A)10	10	109849	109858	SSC
	28	p1	(A)10	10	110303	110312	SSC
	29	p1	(T)14	14	129813	129826	IRA
	1	p1	(T)11	11	1975	1985	LSC
	2	p1	(T)11	11	12928	12938	LSC
A. japonica	3	p1	(A)10	10	13621	13630	LSC
	4	p1	(A)10	10	16852	16861	LSC
	5	p1	(T)11	11	17305	17315	LSC

6	p1	(A)10	10	18675	18684	LSC
7	p1	(T)10	10	23499	23508	LSC
8	p1	(T)10	10	25224	25233	LSC
9	p1	(T)10	10	26317	26326	LSC
10	p1	(T)10	10	28256	28265	LSC
11	p1	(A)10	10	30066	30075	LSC
12	p1	(T)13	13	34937	34949	LSC
13	p1	(A)10	10	35856	35865	LSC
14	p1	(A)10	10	44216	44225	LSC
15	p1	(T)11	11	46303	46313	LSC
16	c	(T)11cattttattttttaattaaaaaataaaatg(A)12	53	46663	46715	LSC
17	p1	(T)15	15	50488	50502	LSC
18	p1	(T)10	10	51472	51481	LSC
19	p1	(T)11	11	54879	54889	LSC
20	p1	(T)11	11	59495	59505	LSC
21	p1	(T)17	17	63375	63391	LSC
22	p1	(A)16	16	65620	65635	LSC
23	p1	(T)11	11	71582	71592	LSC
24	p1	(A)10	10	77952	77961	LSC
25	p1	(T)10	10	78193	78202	LSC
26	p1	(T)13	13	80631	80643	LSC
27	p1	(A)14	14	107663	107676	IRB
28	p1	(A)10	10	109804	109813	SSC
29	p1	(A)10	10	110258	110267	SSC
30	p1	(T)14	14	129725	129738	IRA

Supplementary Table 8. The tandem repeats detected in the plastid genomes of *Atractylodes* species.

Species	Indices	Size(bp)	Repeat number	Start position	Region
A. chinensis	3766--3816	25	2	3766	LSC,tRNA(trnK-UUU)
	12183--12209	13	2.1	12183	LSC,IGS(trnE-UUC_rpoB)
	12439--12502	33	1.9	12439	LSC,IGS(trnE-UUC_rpoB)
	12576--12623	24	2	12576	LSC,IGS(trnE-UUC_rpoB)
	21023--21064	21	2	21023	LSC,CDS(rpoC2)
	25327--25351	12	2.1	25327	LSC,CDS(atpI)
	31893--31928	18	2	31893	LSC,IGS(trnT-GGU_psbD)
	41883--41914	16	2	41883	LSC,IGS(psaA_ycf3)
	47552--47589	18	2.1	47552	LSC,IGS(trnL-UAA_trnF-GAA)
	51204--51233	15	2	51204	LSC,IGS(trnV-UAC_trnM-CAU)
	53964--53995	16	2	53964	LSC,IGS(atpB_rbcL)
	58287--58334	20	2.3	58287	LSC,IGS(accD_psaI)
	62736--62777	19	2.2	62736	LSC,IGS(petA_psbJ)
	62751--62782	16	2	62751	LSC,IGS(petA_psbJ)
	67141--67181	21	2	67141	LSC,CDS(rps18)
	76212--76249	19	2	76212	LSC,CDS(petD)
	88132--88201	21	3.3	88132	IRB,CDS(ycf2)
	90528--90569	18	2.3	90528	IRB,CDS(ycf2)
	106367--106428	32	1.9	106367	IRB,IGS(rrn4.5_rrn5)
	108472--108507	12	3	108472	IRB,CDS(ycf1)
	109155--109245	45	2	109155	SSC,CDS(ycf1)
	109303--109346	21	2.1	109303	SSC,CDS(ycf1)
	111858--111886	12	2.5	111858	SSC,CDS(ycf1)
	111961--112002	21	2	111961	SSC,CDS(ycf1)

	112169--112256	30	2.9	112169	SSC,CDS(ycf1)
	112276--112352	30	2.5	112276	SSC,CDS(ycf1)
	112692--112727	18	2	112692	SSC,CDS(ycf1)
	124582--124613	16	2	124582	SSC,IGS(rpl32_ndhF)
	127063--127108	23	2	127063	SSC,CDS(ndhF)
	129126--129187	32	1.9	129126	IRA,IGS(rrn5_rrn4.5)
	144985--145026	18	2.3	144985	IRA,CDS(ycf2)
	147353--147407	21	2.6	147353	IRA,CDS(ycf2)
	147370--147422	21	2.5	147370	IRA,CDS(ycf2)
	3776--3826	25	2	3776	LSC,tRNA(trnK-UUU)
	12193--12219	13	2.1	12193	LSC,IGS(trnE-UUC_rpoB)
	12449--12512	33	1.9	12449	LSC,IGS(trnE-UUC_rpoB)
	12586--12633	24	2	12586	LSC,IGS(trnE-UUC_rpoB)
	19089--19127	18	2.2	19089	LSC,IGS(rpoC1_rpoC2)
	21050--21091	21	2	21050	LSC,CDS(rpoC2)
	25354--25378	12	2.1	25354	LSC,IGS(atpI_atpH)
	31917--31952	18	2	31917	LSC,IGS(trnT-GGU_psbD)
A. koreana	41911--41942	16	2	41911	LSC,IGS(psaA_ycf3)
	51145--51182	17	2.2	51145	LSC,IGS(ndhC_trnV-UAC)
	52201--52230	15	2	52201	LSC,IGS(trnV-UAC_trnM-CAU)
	59267--59314	20	2.3	59267	LSC,IGS(accD_psaI)
	68111--68151	21	2	68111	LSC,CDS(rps18)
	77182--77219	19	2	77182	LSC,CDS(petD)
	89094--89163	21	3.3	89094	IRB,CDS(ycf2)
	91490--91531	18	2.3	91490	IRB,CDS(ycf2)
	107324--107385	32	1.9	107324	IRB,IGS(rrn4.5_rrn5)

	109430--109465	12	3	109430	SSC,CDS(ycf1)
	109620--109644	9	2.8	109620	SSC,CDS(ycf1)
	109608--109665	21	2.8	109608	SSC,CDS(ycf1)
	110122--110212	45	2	110122	SSC,CDS(ycf1)
	110270--110313	21	2.1	110270	SSC,CDS(ycf1)
	112825--112853	12	2.5	112825	SSC,CDS(ycf1)
	112928--112969	21	2	112928	SSC,CDS(ycf1)
	113130--113217	30	2.9	113130	SSC,CDS(ycf1)
	113237--113307	30	2.6	113237	SSC,CDS(ycf1)
	113647--113682	18	2	113647	SSC,CDS(ycf1)
	124883--124907	12	2.1	124883	SSC,IGS(rpl32_ndhF)
	125553--125584	16	2	125553	SSC,IGS(rpl32_ndhF)
	128031--128076	20	2.3	128031	SSC,CDS(ndhF)
	130095--130156	32	1.9	130095	IRA,IGS(rrn5_rrn4.5)
	145949--145990	18	2.3	145949	IRA,CDS(ycf2)
	148317--148371	21	2.6	148317	IRA,CDS(ycf2)
	148334--148386	21	2.5	148334	IRA,CDS(ycf2)
	3775--3825	25	2	3775	LSC,tRNA(trnK-UUU)
	9833--9877	11	4	9833	LSC,IGS(trnC-GCA_petN)
	9831--9876	23	2	9831	LSC,IGS(trnC-GCA_petN)
	12215--12241	13	2.1	12215	LSC,IGS(trnE-UUC_rpoB)
A. lancea	12471--12534	33	1.9	12471	LSC,IGS(trnE-UUC_rpoB)
	12608--12655	24	2	12608	LSC,IGS(trnE-UUC_rpoB)
	19111--19149	18	2.2	19111	LSC,IGS(rpoC1_rpoC2)
	21072--21113	21	2	21072	LSC,CDS(rpoC2)
	25377--25401	12	2.1	25377	LSC,IGS(atpI_atpH)

31940--31975	18	2	31940	LSC,IGS(trnT-GGU_psbD)
41933--41964	16	2	41933	LSC,IGS(psaA_ycf3)
51169--51206	17	2.2	51169	LSC,IGS(ndhC_trnV-UAC)
52225--52254	15	2	52225	LSC,IGS(trnV-UAC_trnM-CAU)
59291--59338	20	2.3	59291	LSC,IGS(accD_psaI)
68135--68175	21	2	68135	LSC,CDS(rps18)
77206--77243	19	2	77206	LSC,CDS(petD)
89118--89187	21	3.3	89118	IRB,CDS(ycf2)
91514--91555	18	2.3	91514	IRB,CDS(ycf2)
107348--107409	32	1.9	107348	IRB,IGS(rrn4.5_rrn5)
109454--109489	12	3	109454	SSC,CDS(ycf1)
109644--109668	9	2.8	109644	SSC,CDS(ycf1)
109632--109689	21	2.8	109632	SSC,CDS(ycf1)
110146--110236	45	2	110146	SSC,CDS(ycf1)
110294--110337	21	2.1	110294	SSC,CDS(ycf1)
112849--112877	12	2.5	112849	SSC,CDS(ycf1)
112952--112993	21	2	112952	SSC,CDS(ycf1)
113160--113247	30	2.9	113160	SSC,CDS(ycf1)
113267--113337	30	2.6	113267	SSC,CDS(ycf1)
113677--113712	18	2	113677	SSC,CDS(ycf1)
124913--124937	12	2.1	124913	SSC,IGS(rpl32_ndhF)
125583--125614	16	2	125583	SSC,IGS(rpl32_ndhF)
128061--128106	20	2.3	128061	SSC,CDS(ndhF)
130125--130186	32	1.9	130125	IRA,IGS(rrn5_rrn4.5)
145979--146020	18	2.3	145979	IRA,CDS(ycf2)
148347--148401	21	2.6	148347	IRA,CDS(ycf2)

	148364--148416	21	2.5	148364	IRA,CDS(ycf2)
	3764--3814	25	2	3764	LSC,tRNA(trnK-UUU)
	12187--12213	13	2.1	12187	LSC,IGS(trnE-UUC_rpoB)
	12443--12506	33	1.9	12443	LSC,IGS(trnE-UUC_rpoB)
	12580--12627	24	2	12580	LSC,IGS(trnE-UUC_rpoB)
	21026--21067	21	2	21026	LSC,CDS(rpoC2)
	25330--25354	12	2.1	25330	LSC,IGS(atpI_atpH)
	31892--31927	18	2	31892	LSC,IGS(trnT-GGU_psbD)
	41884--41915	16	2	41884	LSC,IGS(psaA_ycf3)
	51125--51162	17	2.2	51125	LSC,IGS(ndhC_trnV-UAC)
	52181--52210	15	2	52181	LSC,IGS(trnV-UAC_trnM-CAU)
	59248--59295	20	2.3	59248	LSC,IGS(accD_psaI)
	68089--68129	21	2	68089	LSC,CDS(rps18)
A. japonica	77161--77198	19	2	77161	LSC,CDS(petD)
	89073--89142	21	3.3	89073	IRB,CDS(ycf2)
	91469--91510	18	2.3	91469	IRB,CDS(ycf2)
	107303--107364	32	1.9	107303	IRB,IGS(rrn4.5_rrn5)
	109409--109444	12	3	109409	SSC,CDS(ycf1)
	109599--109623	9	2.8	109599	SSC,CDS(ycf1)
	109587--109644	21	2.8	109587	SSC,CDS(ycf1)
	110101--110191	45	2	110101	SSC,CDS(ycf1)
	110249--110292	21	2.1	110249	SSC,CDS(ycf1)
	112804--112832	12	2.5	112804	SSC,CDS(ycf1)
	112907--112948	21	2	112907	SSC,CDS(ycf1)
	113115--113202	30	2.9	113115	SSC,CDS(ycf1)
	113222--113292	30	2.6	113222	SSC,CDS(ycf1)

A. macrocephala	113632--113667	18	2	113632	SSC,CDS(ycf1)
	125515--125546	16	2	125515	SSC,IGS(rpl32_ndhF)
	130037--130098	32	1.9	130037	IRA,IGS(rrn5_rrn4.5)
	145891--145932	18	2.3	145891	IRA,CDS(ycf2)
	148259--148313	21	2.6	148259	IRA,CDS(ycf2)
	148276--148328	21	2.5	148276	IRA,CDS(ycf2)
	3764--3814	25	2	3764	LSC,tRNA(trnK-UUU)
	12196--12222	13	2.1	12196	LSC,IGS(trnE-UUC_rpoB)
	12578--12625	24	2	12578	LSC,IGS(trnE-UUC_rpoB)
	19083--19121	18	2.2	19083	LSC,IGS(rpoC1_rpoC2)
	21044--21085	21	2	21044	LSC,CDS(rpoC2)
	25348--25372	12	2.1	25348	LSC,IGS(atpI_atpH)
	31911--31946	18	2	31911	LSC,IGS(trnT-GGU_psbD)
	41909--41940	16	2	41909	LSC,IGS(psaA_ycf3)
	47584--47621	18	2.1	47584	LSC,IGS(trnL-UAA_trnF-GAA)
	51153--51190	17	2.2	51153	LSC,IGS(ndhC_trnV-UAC)
	52213--52242	15	2	52213	LSC,IGS(trnV-UAC_trnM-CAU)
	54973--55004	16	2	54973	LSC,IGS(atpB_rbcL)
	59295--59342	20	2.3	59295	LSC,IGS(accD_psaI)
	68142--68182	21	2	68142	LSC,CDS(rps18)
	77214--77251	19	2	77214	LSC,CDS(petD)
	89131--89200	21	3.3	89131	IRB,CDS(ycf2)
	91527--91568	18	2.3	91527	IRB,CDS(ycf2)
	107367--107428	32	1.9	107367	IRB,IGS(rrn4.5_rrn5)
	109473--109500	12	2.3	109473	SSC,CDS(ycf1)
	109659--109701	9	4.8	109659	SSC,CDS(ycf1)

110174--110264	45	2	110174	SSC,CDS(ycf1)
110322--110365	21	2.1	110322	SSC,CDS(ycf1)
112877--112905	12	2.5	112877	SSC,CDS(ycf1)
112980--113021	21	2	112980	SSC,CDS(ycf1)
113188--113275	30	2.9	113188	SSC,CDS(ycf1)
113295--113371	30	2.5	113295	SSC,CDS(ycf1)
113711--113746	18	2	113711	SSC,CDS(ycf1)
125601--125632	16	2	125601	SSC,IGS(rpl32_ndhF)
130123--130184	32	1.9	130123	IRA,IGS(rrn5_rrn4.5)
145983--146024	18	2.3	145983	IRA,CDS(ycf2)
148351--148405	21	2.6	148351	IRA,CDS(ycf2)
148368--148420	21	2.5	148368	IRA,CDS(ycf2)

Supplementary Table 9. The repeats detected in the plastid genomes of *Atractylodes* species.

Species	The repeat length of the first part	The starting position of the first part	Matching direction	The repeat length of the second part	The starting position of the second part	E-value
A. chinensis	52	46658	P	52	46658	3.22E-22
	48	73398	P	48	73398	8.23E-20
	42	10683	P	42	10683	3.37E-16
	42	43317	P	42	116658	3.37E-16
	35	109154	F	35	109199	5.52E-12
	30	8511	P	30	45077	5.66E-09
	30	112179	F	30	112209	5.66E-09
	27	374	P	27	407	3.62E-07

27	112288	F	27	112318	3.62E-07
26	86924	P	26	86924	1.45E-06
26	86924	F	26	148603	1.45E-06
26	148603	P	26	148603	1.45E-06
25	43318	F	25	97280	5.79E-06
25	43318	P	25	138248	5.79E-06
25	88146	F	25	88167	5.79E-06
25	88146	P	25	147361	5.79E-06
25	88167	P	25	147382	5.79E-06
25	97280	P	25	116674	5.79E-06
25	116674	F	25	138248	5.79E-06
25	147361	F	25	147382	5.79E-06
24	66647	F	24	98450	2.32E-05
24	66647	P	24	137079	2.32E-05
24	90527	F	24	90545	2.32E-05
24	90527	P	24	144984	2.32E-05
24	90545	P	24	145002	2.32E-05
24	144984	F	24	145002	2.32E-05
23	35147	P	23	45019	9.27E-05
23	127062	F	23	127085	9.27E-05
21	8517	F	21	35092	1.48E-03
21	35092	P	21	45080	1.48E-03
21	36282	F	21	65805	1.48E-03
21	58288	F	21	58308	1.48E-03
21	79680	C	21	106725	1.48E-03
21	79680	R	21	128807	1.48E-03

	21	111960	F	21	111981	1.48E-03
	20	9251	P	20	9251	5.93E-03
	20	21113	P	20	21113	5.93E-03
	20	29830	R	20	29830	5.93E-03
	20	41897	F	20	55811	5.93E-03
	20	47898	P	20	51135	5.93E-03
	20	50089	R	20	50089	5.93E-03
	20	51136	P	20	102263	5.93E-03
	20	51136	F	20	133270	5.93E-03
	19	1974	F	19	46656	2.37E-02
	19	4442	F	19	73358	2.37E-02
	19	30238	P	19	36059	2.37E-02
	19	35017	F	19	40880	2.37E-02
	19	47898	F	19	102264	2.37E-02
	19	47898	P	19	133270	2.37E-02
	48	74368	P	48	74368	8.34E-20
	42	10693	P	42	10693	3.41E-16
	42	43345	P	42	117613	3.41E-16
	35	110121	F	35	110166	5.59E-12
	30	8520	P	30	45110	5.73E-09
A. koreana	30	113140	F	30	113170	5.73E-09
	27	375	P	27	408	3.67E-07
	26	87886	P	26	87886	1.47E-06
	26	87886	F	26	149567	1.47E-06
	26	149567	P	26	149567	1.47E-06
	25	43346	F	25	98242	5.87E-06

25	43346	P	25	139212	5.87E-06
25	89108	F	25	89129	5.87E-06
25	89108	P	25	148325	5.87E-06
25	89129	P	25	148346	5.87E-06
25	98242	P	25	117629	5.87E-06
25	117629	F	25	139212	5.87E-06
25	148325	F	25	148346	5.87E-06
24	67617	F	24	99412	2.35E-05
24	67617	P	24	138043	2.35E-05
24	91489	F	24	91507	2.35E-05
24	91489	P	24	145948	2.35E-05
24	91507	P	24	145966	2.35E-05
24	145948	F	24	145966	2.35E-05
23	2005	R	23	2005	9.38E-05
23	35179	P	23	45052	9.38E-05
22	113248	F	22	113278	3.75E-04
21	8526	F	21	35124	1.50E-03
21	19088	F	21	19106	1.50E-03
21	35124	P	21	45113	1.50E-03
21	36310	F	21	66775	1.50E-03
21	51144	F	21	51161	1.50E-03
21	59268	F	21	59288	1.50E-03
21	112927	F	21	112948	1.50E-03
20	9260	P	20	9260	6.01E-03
20	21140	P	20	21140	6.01E-03
20	29855	R	20	29855	6.01E-03

	20	41925	F	20	56791	6.01E-03
	20	47920	P	20	52132	6.01E-03
	20	50105	R	20	50105	6.01E-03
	20	52133	P	20	103220	6.01E-03
	20	52133	F	20	134239	6.01E-03
	20	80651	C	20	107684	6.01E-03
	20	80651	R	20	129775	6.01E-03
	20	128036	F	20	128056	6.01E-03
	19	4452	F	19	74328	2.40E-02
	19	30262	P	19	36092	2.40E-02
	19	34958	P	19	65641	2.40E-02
	19	35049	F	19	40908	2.40E-02
A. lancea	48	74392	P	48	74392	8.34E-20
	42	10715	P	42	10715	3.42E-16
	42	43367	P	42	117643	3.42E-16
	35	110145	F	35	110190	5.60E-12
	30	8519	P	30	45132	5.73E-09
	30	113170	F	30	113200	5.73E-09
	27	375	P	27	408	3.67E-07
	26	87910	P	26	87910	1.47E-06
	26	87910	F	26	149597	1.47E-06
	26	149597	P	26	149597	1.47E-06
	25	43368	F	25	98266	5.87E-06
	25	43368	P	25	139242	5.87E-06
	25	89132	F	25	89153	5.87E-06
	25	89132	P	25	148355	5.87E-06

25	89153	P	25	148376	5.87E-06
25	98266	P	25	117659	5.87E-06
25	117659	F	25	139242	5.87E-06
25	148355	F	25	148376	5.87E-06
24	67641	F	24	99436	2.35E-05
24	67641	P	24	138073	2.35E-05
24	91513	F	24	91531	2.35E-05
24	91513	P	24	145978	2.35E-05
24	91531	P	24	145996	2.35E-05
24	145978	F	24	145996	2.35E-05
23	2004	R	23	2004	9.39E-05
23	9830	F	23	9853	9.39E-05
23	35202	P	23	45074	9.39E-05
22	113278	F	22	113308	3.76E-04
21	8525	F	21	35147	1.50E-03
21	19110	F	21	19128	1.50E-03
21	35147	P	21	45135	1.50E-03
21	36332	F	21	66799	1.50E-03
21	51168	F	21	51185	1.50E-03
21	59292	F	21	59312	1.50E-03
21	112951	F	21	112972	1.50E-03
20	9259	P	20	9259	6.01E-03
20	21162	P	20	21162	6.01E-03
20	29878	R	20	29878	6.01E-03
20	41947	F	20	56815	6.01E-03
20	47944	P	20	52156	6.01E-03

	20	50129	R	20	50129	6.01E-03
	20	52157	P	20	103244	6.01E-03
	20	52157	F	20	134269	6.01E-03
	20	80675	C	20	107708	6.01E-03
	20	80675	R	20	129805	6.01E-03
	20	128066	F	20	128086	6.01E-03
	19	4451	F	19	74352	2.40E-02
	19	30285	P	19	36114	2.40E-02
	19	34981	P	19	65665	2.40E-02
	50	46667	P	50	46667	5.20E-21
	48	74320	P	48	74320	8.32E-20
	42	10689	P	42	10689	3.41E-16
	42	43323	P	42	117565	3.41E-16
	35	110067	F	35	110112	5.59E-12
	30	8517	P	30	45088	5.72E-09
	30	113092	F	30	113122	5.72E-09
	27	375	P	27	408	3.66E-07
A. japonica	26	87841	P	26	87841	1.46E-06
	26	87841	F	26	149473	1.46E-06
	26	149473	P	26	149473	1.46E-06
	25	43324	F	25	98188	5.86E-06
	25	43324	P	25	139127	5.86E-06
	25	89054	F	25	89075	5.86E-06
	25	89054	P	25	148240	5.86E-06
	25	89075	P	25	148261	5.86E-06
	25	98188	P	25	117581	5.86E-06

25	117581	F	25	139127	5.86E-06
25	148240	F	25	148261	5.86E-06
24	67568	F	24	99357	2.34E-05
24	67568	P	24	137959	2.34E-05
24	91435	F	24	91453	2.34E-05
24	91435	P	24	145863	2.34E-05
24	91453	P	24	145881	2.34E-05
24	145863	F	24	145881	2.34E-05
23	2000	R	23	2000	9.37E-05
23	35158	P	23	45030	9.37E-05
22	113200	F	22	113230	3.75E-04
21	8523	F	21	35103	1.50E-03
21	35103	P	21	45091	1.50E-03
21	36288	F	21	66726	1.50E-03
21	51129	F	21	51146	1.50E-03
21	112873	F	21	112894	1.50E-03
20	9257	P	20	9257	6.00E-03
20	21117	P	20	21117	6.00E-03
20	29835	R	20	29835	6.00E-03
20	41903	F	20	56775	6.00E-03
20	47907	P	20	52123	6.00E-03
20	50092	R	20	50092	6.00E-03
20	50494	R	20	51472	6.00E-03
20	52124	P	20	103165	6.00E-03
20	52124	F	20	134155	6.00E-03
20	80604	C	20	107630	6.00E-03

	20	80604	R	20	129690	6.00E-03
	19	4447	F	19	74280	2.40E-02
	19	30241	P	19	36070	2.40E-02
	19	35028	F	19	40886	2.40E-02
	19	47907	F	19	103166	2.40E-02
	19	47907	P	19	134155	2.40E-02
	52	46687	P	52	46687	3.26E-22
	48	74400	P	48	74400	8.34E-20
	42	10696	P	42	10696	3.42E-16
	42	43343	P	42	117677	3.42E-16
	35	110173	F	35	110218	5.60E-12
	30	8517	P	30	45108	5.73E-09
	30	113198	F	30	113228	5.73E-09
	27	375	P	27	408	3.67E-07
	27	113307	F	27	113337	3.67E-07
A. macrocephala	26	87923	P	26	87923	1.47E-06
	26	87923	F	26	149601	1.47E-06
	26	149601	P	26	149601	1.47E-06
	25	43344	F	25	98279	5.87E-06
	25	43344	P	25	139246	5.87E-06
	25	89145	F	25	89166	5.87E-06
	25	89145	P	25	148359	5.87E-06
	25	89166	P	25	148380	5.87E-06
	25	98279	P	25	117693	5.87E-06
	25	117693	F	25	139246	5.87E-06
	25	148359	F	25	148380	5.87E-06

24	67642	F	24	99455	2.35E-05
24	67642	P	24	138071	2.35E-05
24	91526	F	24	91544	2.35E-05
24	91526	P	24	145982	2.35E-05
24	91544	P	24	146000	2.35E-05
24	145982	F	24	146000	2.35E-05
23	35173	P	23	45050	9.39E-05
21	8523	F	21	35118	1.50E-03
21	19082	F	21	19100	1.50E-03
21	35118	P	21	45111	1.50E-03
21	36308	F	21	66800	1.50E-03
21	51152	F	21	51169	1.50E-03
21	59296	F	21	59316	1.50E-03
21	80686	C	21	107726	1.50E-03
21	80686	R	21	129803	1.50E-03
21	112979	F	21	113000	1.50E-03
20	9257	P	20	9257	6.01E-03
20	21134	P	20	21134	6.01E-03
20	29850	R	20	29850	6.01E-03
20	41923	F	20	56820	6.01E-03
20	47930	P	20	52144	6.01E-03
20	50116	R	20	50116	6.01E-03
20	52145	P	20	103263	6.01E-03
20	52145	F	20	134267	6.01E-03
19	4440	F	19	74360	2.40E-02
19	30256	P	19	36085	2.40E-02

	19	34952	P	19	65666	2.40E-02
	19	34967	R	19	34967	2.40E-02
	19	35043	F	19	40906	2.40E-02

Supplementary Table 10. Intraspecific heteroplasmic variations found in *Atractylodes* whole plastid genome.

Sample	Position	Reference	Alternative	Quality
HPAB0001	72515	G	A	108
HPAB0006	34539	C	A	126
HPAB0006	37892	G	T	225
HPAB0006	49175	CTGAAGATTGA	CTGA	29.6821
HPAB0006	65446	A	C	22.934
HPAB0006	72506	GGCAAA	GGCAAAGCAAA	12.8049
HPAB0006	72852	GCTACT	G	96
HPAB0006	73111	T	G	9.32336
HPAB0007	32267	T	G	196
HPAB0007	33324	T	C	221
HPAB0007	78207	ATTTTTTTTTT	ATTTTTTTTTTTTTTT	175

HPAB0011	79384	C	A	26.0502
HPAB0016	74099	T	G	6.51044
HPAB0016	80688	TTTTACGTATTT	TTTT	11.69
HPAB0018	109869	TAAAAAAAAAA	TAAAAAAAAAAAAAAA,TAAAAAAAAAAAAAAAA	4.42804
HPAB0022	107414	T	C	24.6668
HPAB0022	130124	T	C	6.62156
HPAB0024	794	A	C	59
HPAB0024	1252	A	C	6.76493
HPAB0024	6221	GTAAGAATAAGAAT	GTAAGAAT	156
HPAB0024	6424	T	G	77
HPAB0024	6871	AATTTTAATATTTTA	AATTTTA	32.5949
HPAB0024	10808	T	C	79
HPAB0024	11003	T	G	134
HPAB0024	11165	T	G	185
HPAB0024	11193	A	C	178
HPAB0024	11468	A	C	106
HPAB0024	38570	A	G	185

HPAB0024	41512	G	T	14.965
HPAB0024	41532	C	A	38.2342
HPAB0024	42995	AAGCCCAT	A	93
HPAB0024	45151	A	C	155
HPAB0024	45234	TAATGG	T	184
HPAB0024	46710	C	A	5.60274
HPAB0024	47623	G	A	221
HPAB0024	48508	AGAATCCCAATGAATCCC	AGAATCCC	108
HPAB0024	56843	A	C	114
HPAB0024	57783	GAAATA	GAAATAAAATA	24.5956
HPAB0024	66431	C	T	38.8423
HPAB0024	83988	CTATATAT	CTATATATAT	80
HPAB0024	109730	T	G	29.9462
HPAB0024	110366	C	A	222
HPAB0024	123671	A	C	23.9256
HPAB0024	127761	A	C	188
HPAB0025	794	A	C	91

HPAB0025	38570	T	A,G	194
HPAB0025	43394	T	G	56
HPAB0025	45151	A	C	46.8757
HPAB0025	110366	C	A	22.7677
HPAB0025	127761	A	C	67
HPAB0026	1259	A	C	77
HPAB0026	30886	C	A	81
HPAB0026	77983	A	T	3.49728
HPAB0027	5736	TTGGATTCT	TT	70
HPAB0027	11167	T	G	46.5108
HPAB0027	35737	A	C	6.08067
HPAB0027	37885	G	T	228
HPAB0027	41508	C	A	44.7602
HPAB0027	45127	A	C	20.8999
HPAB0027	48356	A	G	43.2729
HPAB0027	65648	C	A	9.30714
HPAB0027	65670	T	C	47.1668

HPAB0027	110339	C	A	175
HPAB0027	127740	A	C	134

Supplementary Table 11. The genes/regions during phylogenetic analysis.

Location	Category	Number of joint genes	Length of alignment	Gene
Nuclear	nrDNA concatenated sequences		5,855	—
Plastid	protein-coding genes	73	61,413	*atpA, atpB, atpE, atpF, atpH, atpI, ccsA, cemA, clpP, infA, matK, ndhA, ndhB, ndhC, ndhD, ndhE, ndhF, ndhG, ndhH, ndhI, ndhJ, ndhK, petA, petB, petD, petL, petN, petG, psaA, psaB, psaI, psaJ, psaC, psbA, psbB, psbC, psbD, psbE, psbF, psbH, psbI, psbJ, psbK, psbM, psbN, psbT, rbcL, rpl14, rpl16, rpl2, rpl20, rpl22, rpl23, rpl32, rpl33, rpl36, rps2, rps3, rps4, rps7, rps8, rps11, rps14, rps15, rps16, rps18, rpoA, rpoB, rpoC1, rpoC2, ycf2, ycf3, ycf4*
	all genes (tRNA, rRNA, protein-coding gene)	95	83,547	*atpA, atpB, atpE, atpF, atpH, atpI, ccsA, cemA, clpP, matK, ndhA, ndhB, ndhC, ndhD, ndhE, ndhF, ndhG, ndhH, ndhI, ndhJ, ndhK, petA, petD, petL, petN, petG, psaA, psaB, psaI, psaJ, psaC, psbA, psbB, psbC, psbD, psbE, psbF, psbH, psbI, psbJ, psbK, psbM, psbN, psbT, rbcL, rpl14, rpl16, rpl2, rpl20, rpl22, rpl23, rpl32, rpl33, rpl36, rps2, rps3, rps4, rps7, rps8, rps11, rps14, rps15, rps16, rps18, rpoA, rpoB, rpoC1, rpoC2, ycf1, ycf2, ycf3, ycf4, rrn5, rrn4.5, rrn23, rrn16, trnC-GCA, trnD-GUC, trnF-GAA, trnH-GUG, trnL-CAA, trnL-UAG, trnN-GUU, trnP-UGG, trnQ-UUG, trnR-ACG, trnR-UCU, trnS-GCU, trnS-GGA, trnS-UGA, trnT-GGU, trnT-UGU, trnV-GAC, trnW-CCA, trnY-GUA*
	IGS regions	88	37,809	*atpA_trnR-UCU, atpE_atpB_rbcL, atpF_atpA, atpH_atpF, atpI_atpH, ccsA_trnL-UAG, cemA_petA,*

				clpP_psbB, ndhA_ndhI, ndhB_rps7, ndhB_trnL-CAA, ndhD_ccsA, ndhE_psaC, ndhG_ndhE, ndhI_ndhG, ndhJ_ndhK, petA_psbJ, petD_rpoA, petG_trnW-CCA, petL_petG, petN_psbM, psaA_ycf3, psaB_psaA, psaC_ndhD, psaI_ycf4, psaJ_rpl33, psbA_trnK-UUU_matK, psbD_psbC_trnS-UGA, psbE_petL, psbH_petB, psbI_trnS-GCU, psbJ_psbL, psbK_psbI, psbM_trnD-GUC, psbN_psbH, psbT_psbN, rpl14_rpl16, rpl16_rps3_rpl22, rpl20_clpP, rpl23_trnI-CAU, rpl2_rpl23, rpl32_ndhF_ycf1, rpl33_rps18, rpl36_infA, rpoA_rps11, rpoB_rpoC1, rpoC1_rpoC2, rpoC2_rps2, rps11_rpl36, rps14_psaB, rps15_ndhH, rps16_trnQ-UUG, rps18_rpl20, rps2_atpI, rps3_rpl22_rps19, rps4_trnT-UGU, rps7_ndhB, rps8_rpl14, rrn16_trnV-GAC, rrn4.5_rrn23, rrn4.5_rrn5, rrn5_rrn4.5, trnC-GCA_petN, trnD-GUC_trnY-GUA, trnF-GAA_ndhJ, trnfM-CAU_rps14, trnH-GUG_psbA, trnI-CAU_rpl23, trnI-CAU_ycf2, trnL-CAA_ndhB, trnL-CAA_ycf2, trnL-UAG_rpl32, trnM-CAU_atpE_atpB, trnN-GUU_trnR-ACG, trnN-GUU_ycf1, trnP-UGG_psaJ, trnQ-UUG_psbK, trnR-UCU_trnG-UCC, trnS-GCU_trnC-GCA, trnS-GGA_rps4, trnT-CGU_psbD_psbC, trnV-GAC_rrn16, trnW-CCA_trnP-TGG, ycf1_rps15, ycf2_trnI-CAU, ycf2_trnL-CAA, ycf3_trnS-GGA, ycf4_cemA
	protein	73	20,393	*atpA, atpB, atpE, atpF, atpH, atpI, ccsA, cemA, clpP, infA, matK, ndhA, ndhB, ndhC, ndhD, ndhE, ndhF, ndhG, ndhH, ndhI, ndhJ, ndhK, petA, petB, petD, petL, petN, petG, psaA, psaB, psaI, psaJ, psaC, psbA, psbB, psbC, psbD, psbE, psbF, psbH, psbI, psbJ, psbK, psbM, psbN, psbT, rbcL, rpl14, rpl16, rpl2, rpl20, rpl22, rpl23, rpl32, rpl33, rpl36, rps2, rps3, rps4, rps7, rps8, rps11, rps14, rps15, rps16, rps18, rpoA, rpoB, rpoC1, rpoC2, ycf2, ycf3, ycf4*
	whole genomes		121,356	—

Supplementary Table 12. D-statistic (ABBA-BABA) result for testing introgression events in genus *Atractylodes*.

P1	P2	P3	Dstatistic	Z-score	p-value	f4-ratio	BBAA	ABBA	BABA
A. chinensis	*A. japonica*	*A. carlinoides*	0.1213	0.6650	0.5060	0.0035	88.3818	1.9606	1.5364
A. chinensis	*A. koreana*	*A. carlinoides*	0.1448	0.9242	0.3554	0.0052	87.7985	2.4682	1.8439
A. chinensis	*A. lancea*	*A. carlinoides*	0.1033	0.7820	0.4342	0.0040	87.4309	2.5673	2.0864
A. chinensis	*A. macrocephala*	*A. carlinoides*	0.2960	1.7652	0.0775	0.0152	83.8697	3.9939	2.1697
A. japonica	*A. koreana*	*A. carlinoides*	0.0604	0.2732	0.7847	0.0017	87.1111	1.7556	1.5556
A. japonica	*A. lancea*	*A. carlinoides*	0.0175	0.1043	0.9169	0.0005	88.8133	1.6467	1.5900
A. japonica	*A. macrocephala*	*A. carlinoides*	0.2877	1.3494	0.1772	0.0117	83.4333	3.1333	1.7333
A. lancea	*A. koreana*	*A. carlinoides*	0.0341	0.2064	0.8365	0.0012	87.3117	2.1717	2.0283
A. koreana	*A. macrocephala*	*A. carlinoides*	0.2134	0.9382	0.3481	0.0101	84.0222	3.4111	2.2111
A. lancea	*A. macrocephala*	*A. carlinoides*	0.2350	1.2544	0.2097	0.0113	83.7200	3.5300	2.1867
A. chinensis	*A. japonica*	*A. koreana*	0.0038	0.0309	0.9753	0.0058	4.1914	3.3258	3.3005
A. lancea	*A. japonica*	*A. chinensis*	0.2380	2.3644	0.0181	0.4437	4.7055	3.9033	2.4024
A. chinensis	*A. japonica*	*A. macrocephala*	0.1040	0.5375	0.5909	0.0125	6.9727	2.2515	1.8273
A. chinensis	*A. koreana*	*A. lancea*	0.0533	0.7069	0.4796	0.1843	4.3338	4.1435	3.7238
A. chinensis	*A. koreana*	*A. macrocephala*	0.1245	1.1982	0.2308	0.0216	6.8399	3.3207	2.5854

A. chinensis	*A. lancea*	*A. macrocephala*	0.0556	0.4483	0.6539	0.0092	6.3133	2.9830	2.6688
A. lancea	*A. japonica*	*A. koreana*	0.0335	0.3295	0.7417	0.0500	4.9972	3.3294	3.1139
A. japonica	*A. koreana*	*A. macrocephala*	0.0633	0.3157	0.7522	0.0093	6.1556	2.6111	2.3000
A. lancea	*A. japonica*	*A. macrocephala*	0.0288	0.1905	0.8489	0.0033	7.4867	1.9633	1.8533
A. lancea	*A. koreana*	*A. macrocephala*	0.0707	0.5461	0.5850	0.0125	6.5161	3.1872	2.7661
